# Distinct histone methylation and transcription profiles are established during the development of cellular quiescence in yeast

**DOI:** 10.1186/s12864-017-3509-9

**Published:** 2017-01-26

**Authors:** Conor P. Young, Cory Hillyer, Karsten Hokamp, Darren J. Fitzpatrick, Nikifor K. Konstantinov, Jacqueline S. Welty, Scott A. Ness, Margaret Werner-Washburne, Alastair B. Fleming, Mary Ann Osley

**Affiliations:** 1 Department of Microbiology, Moyne Institute of Preventive Medicine, School of Genetics and Microbiology, University of Dublin, Trinity College Dublin, Dublin, Ireland; 2 Department of Microbiology and Molecular Genetics, University of New Mexico School of Medicine, Albuquerque, NM USA; 3 Smurfit Institute of Genetics, School of Genetics and Microbiology, University of Dublin, Trinity College Dublin, Dublin, Ireland; 4 University of New Mexico School of Medicine, Albuquerque, NM USA; 5 Department of Biology, University of New Mexico, Albuquerque, NM USA; 6 Division of Molecular Medicine, Department of Internal Medicine, University of New Mexico School of Medicine, Albuquerque, NM USA

**Keywords:** Cellular quiescence, Histone methylation, Transcription

## Abstract

**Background:**

Quiescent cells have a low level of gene activity compared to growing cells. Using a yeast model for cellular quiescence, we defined the genome-wide profiles of three species of histone methylation associated with active transcription between growing and quiescent cells, and correlated these profiles with the presence of RNA polymerase II and transcripts.

**Results:**

Quiescent cells retained histone methylations normally associated with transcriptionally active chromatin and had many transcripts in common with growing cells. Quiescent cells also contained significant levels of RNA polymerase II, but only low levels of the canonical initiating and elongating forms of the polymerase. The RNA polymerase II associated with genes in quiescent cells displayed a distinct occupancy profile compared to its pattern of occupancy across genes in actively growing cells. Although transcription is generally repressed in quiescent cells, analysis of individual genes identified a period of active transcription during the development of quiescence.

**Conclusions:**

The data suggest that the transcript profile and histone methylation marks in quiescent cells were established both in growing cells and during the development of quiescence and then retained in these cells. Together, this might ensure that quiescent cells can rapidly adapt to a changing environment to resume growth.

**Electronic supplementary material:**

The online version of this article (doi:10.1186/s12864-017-3509-9) contains supplementary material, which is available to authorized users.

## Background

When cells are deprived of essential nutrients, they exit the cell cycle and enter the G0 phase, a reversible state of cellular quiescence [1]. Quiescence ensures survival in the face of limiting nutrients through the establishment of a stable, non-growing state and is a property of embryonic stem (ES) cells as well as many adult stem cells, where it maintains a reservoir of cells to restore tissue integrity [2–4]. The key features of quiescent G0 cells are the absence of DNA replication, a low level of gene transcription, reduced RNA content, high respiratory capacity, and the ability to re-enter the cell cycle when conditions become favorable [1]. Characterization of the molecular signatures of quiescent stem cells has revealed several distinguishing features of the genomes of these cells. Transcript profiling has shown that RNA polymerase II occupies promoter regions but not gene bodies in quiescent ES cells, consistent with promoter-proximal pausing [5]. The serine 2 (Ser2) phosphorylated form of the RNA polymerase II C-terminal domain (CTD) is absent in quiescent adult stem cells, indicative of the absence of transcription elongation in these cells [6]. Epigenetic profiling has revealed that quiescent ES cells contain a bivalent chromatin domain in which an activating histone modification, H3 lysine 4 tri-methylation (H3K4me3), and a repressing modification, H3 lysine K27 tri-methylation (H3K27me3), are simultaneously present at the promoters of many genes important for lineage specification [7, 8]. In quiescent adult stem cells, the promoters of many genes, even those not being actively transcribed, are marked with H3K4me3, which is considered a permissive state for transcription [9, 10]. Collectively, these studies have promoted the hypothesis that quiescence represents a poised state that enables rapid gene activation when G0 cells are stimulated to resume proliferation or to differentiate.

The functional relationship between the transcription and epigenetic states of quiescent cells is not well defined. It is also not known how these states are established during the development of quiescent cells. We addressed these issues using a budding yeast model of cellular quiescence [11–13]. When glucose is naturally depleted from *Saccharomyces cerevisiae* cells growing in rich medium, the cells undergo one slow doubling before arresting in stationary phase [11, 13]. Although stationary phase cells have long been considered to be quiescent, they are actually comprised of two distinct cell populations – a stable, quiescent population of non-growing G0 cells that can rapidly re-enter the cell cycle when glucose is restored, and an unstable, nonquiescent population that undergoes necrosis and apoptosis [14]. The development of a technique to separate quiescent cells from nonquiescent cells has enabled the characterization of pure populations of both mature and developing quiescent cells. This has allowed the analysis of the transcription and epigenetic profiles of quiescent cells to be defined over time in the absence of interference from the nonquiescent cell population [14–16].

Similar to quiescent adult stem cells, quiescent yeast cells are non-proliferating and have low transcriptional activity [15–19]. The low level of transcription is associated with global decreases in the levels of histone acetylation that occurs during the entry into quiescence, in part through the recruitment of the Rpd3 histone deacetylase to almost half of the genes repressed by the quiescence-specific transcription factors, Xbp1 and Stb3 [16]. In this study, we investigated histone methylation associated with actively transcribed genes – methylation of histone H3 on lysines 4, 36, and 79 – to determine if these modifications were also decreased in purified quiescent cells [20–23]. Despite the general shut down of transcription, quiescent cells retained high levels of the tri-methylated H3 species, H3K4me3, H3K36me3, and H3K79me3, with similar numbers of genes carrying these modifications in growing and quiescent cells. Quiescent cells also had high global levels of RNA polymerase II (RNAP II), but only low levels of the initiating and elongating forms of RNAP II, consistent with their reduced transcriptional activity. However, a significant number of transcripts were detected in quiescent cells, with many of the transcripts representing RNAs that are made in log cells and stored in RNA-protein complexes in quiescent cells [17]. By following the transcription and histone methylation patterns on individual genes during the formation of quiescent cells, we found that at some genes the transcript and epigenetic profiles in mature quiescent cells were inherited from growing cells, while at other genes these profiles were established during early stages in the development of quiescent cells and then retained in this cell population. Analysis of mutants deficient for histone methylation also suggests that specific methylation marks play distinct roles in the establishment and maintenance of the quiescent state.

## Results

### Global Levels of histone methylation during the formation of quiescent cells

**Fig. 1:**
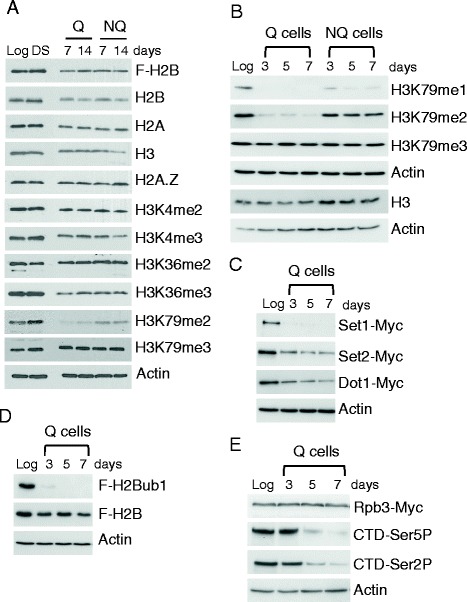
Histone modifications in growing and quiescent cells. **a**. TCA lysates were prepared from cells in log phase (Log), at the diauxic shift (DS), and in purified populations of quiescent (Q) and nonquiescent (NQ) cells isolated 7 and 14 days after culture inoculation. Western blots were probed with antibodies against histones and methylated histones. An empty lane between the DS samples and day-7 Q samples was removed from the image. **b** Western blots were probed with antibodies against H3K79me1, H3K79me2, and H3K79me3 in lysates from log cells and purified populations of Q and NQ cells isolated 3, 5, and 7 days after culture inoculation. **c**-e Lysates were prepared from log cells and purified Q cells isolated at 3, 5, and 7 days after culture inoculation and western blots were probed with (**c**) anti-Myc antibodies to detect Set1, Set2, and Dot1; (**d**) anti-Flag antibodies to detect H2B and monoubiquitinated H2B (H2Bub1); and (**e**) antibodies that recognize unmodified RNA polymerase II (Rpb3-Myc) and the serine 5 (CTD-Ser5P) and serine 2 (CTD-Ser2P) phosphorylated forms of the RNAP II CTD. Actin served as a loading control in all blots, and the images represent the results from a single time-course experiment. A representative Actin blot is shown in panel **a**

To investigate the histone methylation landscape during the development of quiescence, we grew yeast cells in glucose-containing rich medium and prepared lysates from cells in mid-log phase (growing), at the point of glucose depletion (diauxic shift), and from purified populations of quiescent (Q) and nonquiescent (NQ) cells isolated 7 and 14 days after inoculation [14]. Western blot analysis was then performed with antibodies against histones and histones modified by methylation (Fig. 1a). There was no change in the abundance of the core (H2A, H2B, H3) or variant (H2A.Z) histones between log cells and cells at the diauxic shift, and small decreases in the abundance of these proteins between diauxie and Q or NQ cells. In contrast to the global decrease in the levels of histone acetylation in both a mixed population of stationary phase cells [24] and in isolated quiescent cells (Additional file 1: Figure S1E) [16], the levels of H3K4 di-methylation (H3K4me2) and H3K36 di- and tri-methylation (H3K36me2, me3) were similar between log cells and purified Q and NQ cells (Fig. 1a; Additional file 1: Figure S1F). This is consistent with the report that these modified histones were present at high levels in unseparated stationary phase cells formed upon severe nutrient deprivation [24]. However, the levels of H3K4 tri-methylation (H3K4me3) decreased by about 50% in both Q and NQ cells and there was a significant loss of both H3K79 mono- and di-methylation (H3K79me1, me2) in Q cells compared to NQ cells (Fig. 1a, b; Additional file 1: Figure S1F). In contrast, the levels of H3K79 trimethylation (H3K79me3) increased at the diauxic shift and remained at a high level in both Q and NQ cells (Fig. 1a, b: Additional file 1: Figure S1F). Thus, while Q cells are distinguished from log cells by their loss of histone acetylation, they are similar to both log and diauxic cells in their retention of most forms of H3 methylation. Q cells are further differentiated from both log and NQ cells by their selective loss of H3K79me1 and me2.

To investigate when the levels of H3K79me1 and me2 began to decrease as Q cells developed, we probed lysates prepared from pure populations of Q and NQ cells that were isolated at 3, 5, and 7 days after culture inoculation (Fig. 1b). At day 3, cells had completed the final cell doubling that occurs after glucose depletion, and day 5 represented the midpoint in the development of the fully quiescent state (Additional file 1: Figure S1A) [14, 25]. H3K79me1 and H3K79me2 were present at very low levels in day-3 Q cells, while H3K79me3 levels did not significantly change in these cells throughout the sampling period (Fig. 1b). This phenotype appears to be a unique property of Q cells, as the levels of H3K79me3, H3K79me2, and to a lesser extent, H3K79me1, remained relatively unchanged in NQ cells isolated during the same time course (Fig. 1b). The loss of the mono- and di-methylation states in Q cells was specific to H3K79, as H3K4me1, H3K4me2, and H3K4me3 were present throughout Q cell development (Additional file 1: Figure S1B). Although the levels of Dot1, the H3K79 modifying enzyme, decreased in day-3 Q cells (Fig. 1c), the selective loss of H3K79me1 and H3K79me2 in Q cells suggests that either a Q-specific H3K79me1 and H3K79me2 demethylase was activated, or the activity of Dot1 was altered to promote only H3K79 tri-methylation. Alternatively, nucleosomes that contained H3K79me1 or me2 could have been selectively replaced with unmodified H3.

H3K4 and H3K79 methylation are regulated by H2B monoubiquitylation (H2Bub1), and H3K4 and H3K36 methylation are regulated by phosphorylation of the RNA polymerase II (RNAP II) C-terminal domain (CTD) on serine 5 (Ser 5) or serine 2 (Ser2), which mediate transcription initiation and elongation, respectively [21, 26–30]. These modified forms of RNAP II recruit the lysine methyltransferases, Set1 and Set2, to chromatin and thus couple H3K4 and H3K36 methylation to on-going transcription. H2Bub1 disappeared at diauxie in response to glucose depletion, as previously reported, and was absent in both Q and NQ cells isolated after this event (Fig. 1d and data not shown) [31]. RNAP II (Rpb3-Myc), TBP, and Mediator subunit, Srb4, were present in both log and 7-day Q cells, consistent with retention of the pre-initiation complex (Additional file 1: Figure S1C, D) [16, 32]. However, these same Q cells contained only low levels of both CTD Ser5-P and CTD Ser2-P modified RNAP II compared to log cells (Additional file 1: Figure S1C). This result is similar to what has been reported in unseparated stationary phase cells [32], and supports the conclusion that both transcription initiation and elongation are generally repressed in mature Q cells. To determine when Ser5 and Ser2 CTD phosphorylation declined during Q cell development, we followed the two forms of RNAP II in purified Q cells isolated 3, 5, and 7 days after culture inoculation (Fig. 1e). Surprisingly, both RNAP II Ser5 and Ser2 phosphorylation remained at high levels in day-3 Q cells before decreasing in abundance by day 5. In contrast, Set1 was not detected in day-3 Q cells, and Set2 was present at reduced levels in Q cells from day-3 through day-7. (Fig. 1c). This suggests that H3K4me2/me3 and H3K36me2/me3 were established on chromatin by the coupled activity of RNAP II and the two histone methyltransferases shortly before or soon after diauxie, and then retained in Q cells in the absence of their usual regulatory signals.

### Genome-wide localization of histone methylation and RNAP II in quiescent cells

**Fig. 2:**
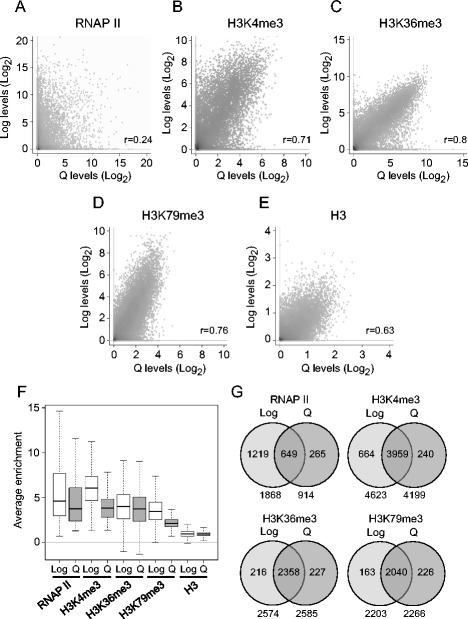
Genome-wide distribution of RNAP II and H3 methylations in growing and quiescent cells. **a**-**e** Scatter plots showing correlation of RNAP II (**a**), H3K4me3 (**b**), H3K36me3 (**c**), H3K79me3 (**d**), and H3 (**e**) signals across the genome of log and 7-day Q cells. **f** Box plots showing the average enrichment of RNAP II, H3K4me3, H3K36me3, H3K79me3 and H3 on genes in log and Q cells. **g** Venn diagrams identifying genes marked with RNAP II and the three H3 PTMS only in log cells, only in Q cells, and in both log and Q cells

We defined the genome-wide distributions of H3K4me3, H3K36me3, H3K79me3, and RNAP II on chromatin isolated from 7-day Q cells and compared the patterns to those found in log cells. A genome browser view of PTM occupancy across yeast chromosome III showed similar patterns in log and Q cells, with the majority of the PTM binding sites associated with gene coding regions (Additional file 1: Figure S2A). Moreover, an unbiased analysis across the genome revealed a strong correlation in all three H3 methylation profiles, as well as in the unmodified H3 profile, between the two cell types (Fig. 2b-e). This is in contrast to the weak correlation in the H3K36me3 profile between log cells and unseparated stationary phase cells formed following complete nutrient deprivation after their transfer into water [24]. Consistent with the genome-wide PTM profiles, log and Q cells contained similar numbers of gene binding sites for the three H3 modifications with each individual modification enriched on at least 35% of all identified *S. cerevisiae* ORFs (Additional file 1: Figure S2B). However, with the exception of H3K36me3, the average enrichment of H3K4me3 and H3K79me3 on genes in Q cells was lower than in log cells (Fig. 2f). This was not the result of decreased H3 occupancy in quiescent cells, as similar levels of H3 were present on chromatin in both log and Q cells (Fig. 2f).

In contrast to the genomic profiles of the H3 PTMs, the RNAP II genome-wide occupancy patterns in log and Q cells were different in several respects. First, as previously reported, there were significant differences in the distribution of the polymerase between the two cell types (Additional file 1: Figure S2A), and a reduced correlation in the RNAP II profile between log and Q cells (Fig. 2a) [16]. Second, Q cells contained over 900 fewer RNAP II gene binding sites than log cells (1868 vs. 914) (Additional file 1: Figure S2B). Moreover, the mean enrichment of RNAP II on genes was also reduced in Q cells, (Fig. 2f). Together, the results show that while transcription is globally repressed in quiescent cells, these cells retained some features normally associated with transcriptionally active chromatin.

### Histone methylation and RNAP II occupancy profiles on genes in quiescent cells

We assigned the genomic H3 methylation and RNAP II binding sites to three subsets of genes: genes marked only in log cells (Log), only in Q cells (Q), and in both log and Q cells (Common) (Additional file 1: Figure S5A-D; ChIP-chip data processing). The majority of genes enriched with each H3 modification were common to both cell types, with a much smaller number of genes marked only in Q or only in log cells (Fig. 2g; Additional file 2: Table S2). In contrast, a larger percentage of the RNAP II marked genes were log cell specific (65%) (Fig. 2g; Additional file 2: Table S2). The log-only H3 methylation and RNAP II marked genes were predominantly associated with genes involved in cell growth and division, while the Q-only marked genes have roles in the cellular response to stress, protein catabolism, and energy production (Additional file 1: Figure S2C; Additional file 3: Table S3). These GO categories suggest that the PTMs and RNAP II were associated with genes that are actively transcribed in each cell type or have the potential to be transcribed.

**Fig. 3:**
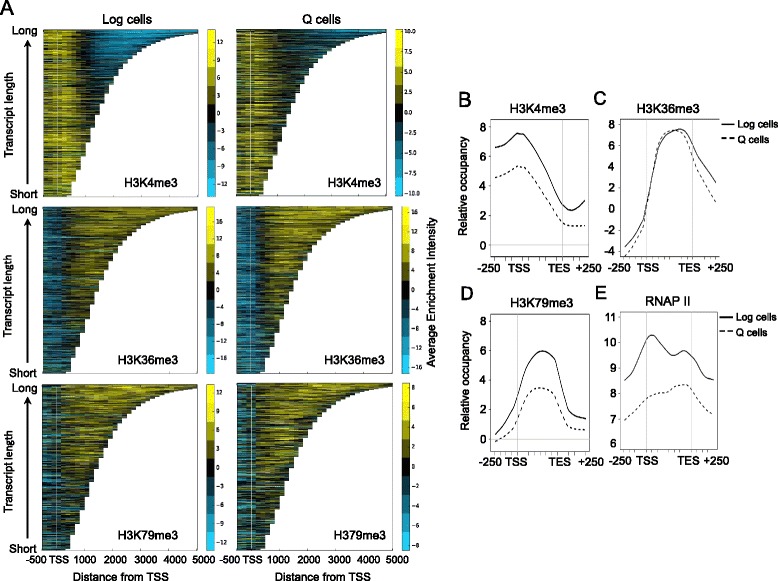
Distribution of H3 methylations and RNAP II on genes in growing and quiescent cells. **a** The CHROMATRA tool was used to visualize the enrichment of H3K4me3, H3K36me3, and H3K79me3 in log and 7-day Q cells across all transcripts sorted by their length (bp). TSS represents transcription start site, and positions upstream (−500 bp) and downstream (5000 bp) of the TSS are indicated. **b**-**e** Averaged gene analysis of the top 25% binding sites on genes for H3K4me3 (**b**); H3K36me3 (**c**); H3K79me3 (**d**); and RNAP II (**e**), in Log and 7-day Q cells relative to the TSS and transcription termination sites (TES), with positions upstream (−250 bp) and downstream (+250 bp) of these sites indicated

Having observed that a significant number of genes were enriched for each H3 PTM in log and Q cells, we investigated whether the gene-association patterns of the modifications were different in the two cell types. We first visualized the distribution of the H3 PTMs across all genes in log or Q cells as a function of transcript length (Fig. 3a) [33, 34]. In both cell types, H3K4me3 was present around the transcription start site (TSS) and H3K36me3 and H3K79me3 were enriched in gene bodies, consistent with previously observed patterns in growing cells [35, 36]. To further assess the gene-association patterns of the PTMs, we examined the averaged H3 methylation profiles on genes in log or Q cells (Fig. 3b-d)*.* In both log and Q cells, H3K4me3 was enriched around the promoter and 5’ coding region, while H3K36me3 and H3K79me3 occupancy was restricted to the coding region. The analysis also showed a reduced overall occupancy of both H3K4me3 and H3K79me3 on genes in Q cells, which was also observed in the unbiased genome-wide analysis (Fig. 2f). While the reduction in H3K4me3 occupancy on genes in Q cells correlated with the reduced global levels of this H3 mark, this was not the case for H3K79me3. Importantly, there was no redistribution of the H3 marks to non-canonical locations within genes, or to intergenic regions, during the development of Q cells.

In contrast to the distribution of the PTMs, the enrichment of RNAPII on genes was strikingly different between log and Q cells (Fig. 3e). In log cells, the gene-averaged Rpb3-Myc profile showed RNAP II enrichment throughout the coding region and into the 3’ UTR, with peak occupancy occurring around the transcription start site. In Q cells, RNAP II was also enriched throughout the coding region but with several distinct differences: promoter proximal RNAP II enrichment was absent, RNAP II enrichment increased at the 3’ transcription end site (TES), and the overall levels of gene-averaged RNAP II were lower in quiescent cells compared to log cells (Fig. 3e; Fig. 2f).

To investigate the gene association patterns of RNAP II in more detail, we defined the averaged distribution of RNAP II on genes in the three gene categories (log, Q, and common) in log and Q cells (Additional file 1: Figure S3A, B). In log cells, RNAP II association with Q-specific genes was significantly reduced, and similarly, there was minimal association of the polymerase with log-specific genes in quiescent cells. These results are consistent with the repression of transcription of the two groups of genes in cells where they should not be expressed. We divided the common genes into two categories: those with RNAP II enriched more highly in log cells, and those with RNAP II enriched more highly in Q cells. Despite the different levels of enrichment, both categories of common genes showed similar patterns of RNAP II distribution (Additional file 1: Figure S3A, B). In log cells, RNAP II was enriched on these genes in a pattern similar to that seen on log-specific genes (Additional file 1: Figure S3A, B left panels). However, in quiescent cells, the pattern of RNAP II association with the common genes differed in two respects from that associated with log-specific genes (Additional file 1: Figure S3A, B right panels). First, the polymerase remained associated with the common genes. Second, the pattern of RNAP II association with these genes was now similar to the pattern seen on Q-specific genes, showing a higher occupancy at the 3’ gene region. These data support the conclusion that there was a shift in the properties of RNAP II on the common genes during the development of quiescent cells, which could reflect changes in the dynamics of polymerase initiation and processivity during this period.

It was previously reported that RNAP II was present at a significant number of intergenic regions (IGRs) in unseparated stationary phase cells, and that this association might poise a subset of genes for rapid activation upon exit from quiescence [32]. We re-examined this issue in purified 7-day Q cells by measuring the distribution of Rpb3-Myc on IGRs that separate divergently transcribed genes (Additional file 4: Table S4). The data showed that 14.1% of RNAP II binding sites in Q cells were associated with IGRs, versus 7.9% of these sites in log cells. In contrast, a higher percentage of gene containing regions (GCRs) were associated with RNAP II in log cells (72.4%) than in Q cells (51.6%). Interestingly, the majority of IGRs with bound RNAP II in both log and 7-day Q cells (77, log; 74, Q) contained CUTs, SUTs, mRNAs involved in yeast transposon assembly and function, or transcripts from the long terminal repeat (LTR) of Ty elements (Additional file 1: Figure S3C; Additional file 4: Table S4). Moreover, the IGR regions occupied by RNAP II in Q cells were not present upstream of the so-called “rapid exit” category of genes [32], although they did include several genes with roles in chromatin remodeling and modification. The presence of RNAP II in the promoters of this latter group of genes might play a role in resolving the compacted genome of Q cells upon their re-entry into the cell cycle [37, 38]. Together, the data suggest that RNAP II is not poised at the majority of protein-encoding genes in Q cells and that its presence in intergenic regions is related to the production of noncoding RNAs.

### The transcriptome of quiescent cells

**Fig. 4:**
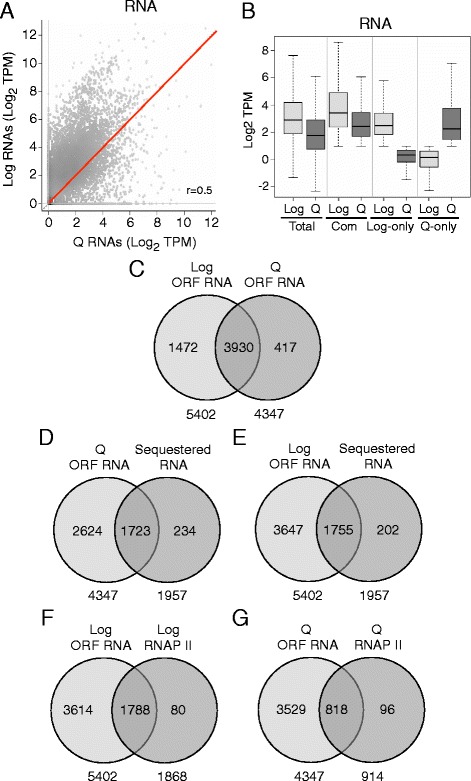
RNA content of growing and quiescent cells. **a** Scatter plot comparing transcript abundance between log and 7-day Q cells. **b** Box plots of transcript abundance for all genes, genes common to log and Q cells, and log-only and Q-only genes in log and Q cells. **c** Venn analysis showing the number of ORF transcripts that are unique and common in each cell type. **d**-**g** Venn analysis showing the number of ORF RNAs associated with RNAs classified as sequestered RNAs in Q cells (**d**) and log cells (**e**), and the number of ORFs associated with RNAP II and transcripts in log cells (**f**) and Q cells (**g**)

While there is a large reduction in active transcription in mature quiescent cells, these cells have also been reported to contain a significant cohort of stored or sequestered RNAs important for their survival [17]. To examine this issue in more detail, we defined the transcriptomes of both 7-day Q cells and log cells using RNA-seq. As previously reported, global RNA levels were significantly lower in quiescent cells than in log cells, and both the overall number and abundance of specific transcripts were also reduced in Q cells (Fig. 4a, b; Additional file 1: Figure S4A) [16]. However, many RNAs could still be detected in Q cells (5105), although these cells contained 1100 fewer transcripts than log cells (6205) (Additional file 1: Figure S4A). A majority of the RNAs in both cell types were produced by RNAP II and fell into four main categories: coding region (ORF) RNAs, cryptic unstable transcripts (CUTs), stable unannotated transcripts (SUTs), and small nuclear RNAs (snRNAs) (Additional file 1: Figure S4A; Additional file 5: Table S5) [39]. Most of the RNAs were associated with ORFs (Additional file 1: Figure S4A) and represented full-length transcripts, suggesting that they arose from a complete round of transcription. The noncoding CUT RNAs predominated in log cells and SUT RNAs were more abundant in Q cells, while snRNAs were present in both cell types without bias (Additional file 1: Figure S4A). Venn analysis showed that the majority of the ORF RNAs were found in both cell types (3930), with <10% (417) of the Q cell RNAs specific to quiescent cells, and 27% (1472) of the RNAs in log cells specific to this cell type (Fig. 4c; Additional file 6: Table S6). As seen for all RNAs, the median level of ORF RNAs common to both cell types was lower in Q cells than in log cells, while a more profound difference in RNA levels was seen for transcripts unique to either log or Q cells in quiescent or growing cells, respectively (Fig. 4b).

Approximately 40% of the Q cell ORF transcripts (1723) corresponded to the RNAs that have been reported to be sequestered in protein-RNA complexes in quiescent cells [17], and these same RNAs were also present in the log cell ORF population (1755) (Fig. 4d, e). We asked if there was a correlation between genes marked with RNAP II and the presence of transcripts in the two cell types. There was a stronger correlation between RNAP II and transcripts in log cells than in Q cells. In log cells, 33% (1788) of ORF RNAs were associated with genes that had bound RNAP II, while in Q cells 19% (818) of ORF RNAs showed a similar association (Fig. 4f, g). Together, the results support the view that a significant fraction of the RNA population in Q cells was transcribed from genes in log cells and stored in Q cells, while a smaller fraction of the Q cell RNAs resulted from active transcription during the development of Q cells. In support of active transcription in Q cells, Q cell-specific RNAs were associated with genes involved with cell survival, including the response to stress, protein catabolism, and autophagy (Additional file 1: Figure S4B; Additional file 7: Table S7). In contrast, ORF RNAs found only in log cells encoded proteins dedicated to cell growth and division (Additional file 1: Figure S4B; Additional file 7: Table S7).

We queried genes associated with RNA only in log cells or only in Q cells for the occupancy of RNAP II and the three H3 PTMs by deriving average gene profiles (Additional file 1: Figure S3D-G). The PTM profiles were very similar to the profiles derived for genes in the absence of their transcription status (Additional file 1: Figure S3D-E; Fig. 3b-d). However, the RNAP II profile on Q genes did not show the canonical 5’ peak characteristic of active genes in log cells, although high levels of polymerase were associated with the coding region of these Q genes (Additional file 1: Figure S3G; Fig. 3e). This suggests that these Q genes underwent an earlier period of high transcriptional activity during their development, and that after the initiation of transcription was repressed, RNAP II was retained across the coding region in mature Q cells.

### Correlation between RNAP II and H3 methylation in quiescent cells

**Fig. 5:**
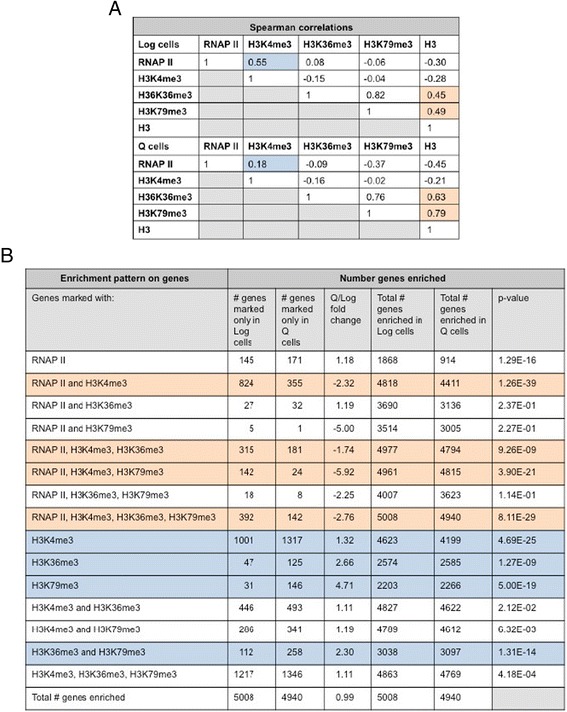
Correlation of RNAP II and H3 methylation signals on genes between log and quiescent cells. **a** Spearman correlations were derived from scatter plots comparing RNAP II, H3K4me3, H3K36me3, H3K79me3, and H3 signals across the genome of log and 7-day Q cells. **b** Number of genes in log and 7-day Q cells co-enriched for RNAP II, H3K4me3, H3K36me3, and H3K79me3 were identified from 4-way Venn analysis. P-values represent Fisher’s exact test

The finding that many genes in Q cells retained RNAP II, H3K4me3, H3K36me3, or H3K79me3 raised the question of whether there was a correlation between the presence of RNAP II and the presence of the three H3 PTMs on genes in these cells. An unbiased analysis of these factors across the genome in log cells showed a strong correlation between the presence of RNAP II and H3K4me3, but little to no correlation between RNAP II and H3K36me3 or H3K79me3 (Fig. 5a). In Q cells, there was a much weaker correlation between the presence of RNAP II and H3K4me3, and similar to log cells, there was no correlation between RNAP II and H3K36me3 or H3K79me3 (Fig. 5a). Another significant difference between log and Q cells was seen in the correlation between H3K36me3, H3K79me3 and histone H3, with Q cells showing a stronger correlation between the PTMs and H3 than log cells (Fig. 5a).

A similar scenario was seen when genes were specifically queried for the co-enrichment of RNAP II and the three species of H3 methylation (Fig. 5b). Significantly, more genes in log cells were co-enriched for RNAP II and combinations of H3K4me3, H3K36me3, and H3K79me3 than in quiescent cells, while more genes in Q cells were co-enriched for the PTMs in the absence of RNAP II. Together, the data support the view that a large fraction of the H3 marks on genes in mature Q cells were initially established in log phase cells or during the development of quiescence, and then retained.

### Gene expression and histone methylation patterns on individual genes during the development of quiescent cells

Because pure populations of quiescent cells can be isolated shortly after diauxie [14], this allowed us to investigate gene expression and histone methylation patterns in Q cells during their development. We isolated Q cells at two-day intervals after diauxie (3, 5, and 7 days after culture inoculation), and examined a subset of genes for the levels of transcripts they produced, and the occupancy of RNAP II and the three H3 PTMs. We used three groups of genes for analysis: genes associated with transcripts present only in log cells, only in Q cells, or in both log and Q cells. Distinct patterns emerged for the different classes of genes.

**Fig. 6:**
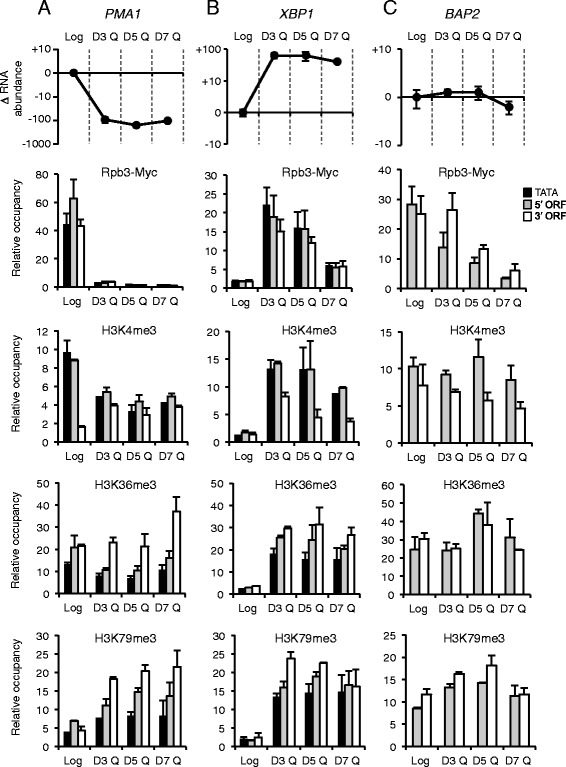
Transcript, RNAP II, and H3 methylation profiles of individual genes during the development of quiescence. Log and Q cells isolated at 3, 5, and 7 days after culture inoculation were analyzed for transcript levels and Rpb3-Myc, H3K4me3, H3K36me3, and H3K79me3 occupancy at (**a**) *PMA1*, a log cell expressed gene; (**b**) *XBP1*, a Q cell expressed gene; and (**c**) *BAP2*, a gene expressed in both cell types. Transcript levels represent the change in RNA abundance relative to levels in log cells. Rpb3-Myc and H3 modification occupancies were determined at the TATA and 5’ or 3’ ORF regions of each gene. Relative occupancy represents the IP/Input at each position relative to IP/Input at *TELV* (Rpb3-Myc) or to H3 IP/Input (H3K4me3, H3K36me3, H3K79me3). The data represent the average with STD of 2 biological replicates

The log-only expressed genes, *PMA1* and *CLN3,* were associated with high levels of RNAP II, H3K4me3, H3K36me3, H3K79me3, and RNA in log cells, consistent with their active transcription (Fig. 6a; Additional file 1: Figure S6A and S7D). However, in Q cells isolated 3 days after inoculation (D3 Q), the levels of RNA and RNAP II associated with these genes had decreased to background levels. The levels of the RNAP II Ser5- and Ser2-phosphorylated CTD species (CTD-Ser5P and CTD-Ser2-P) also decreased at the log-only genes in the developing Q cells during the same period, reflecting the shutdown of their transcription (Additional file 1: Figure S7A). Interestingly, all three H3 PTMs persisted at these log-only expressed genes during Q cell development, albeit to varying extents: H3K4me3 occupancy decreased, H3K36me3 levels remained unchanged, and H3K79me3 levels increased, while no changes were noted for H3 occupancy (Fig. 6a; Additional file 1: Figure S6A, S7B,D). These data suggest that the marks were initially established on these genes during a period of active transcription in log cells and then retained in Q cells following the cessation of transcription.

The Q-only expressed genes, *XBP1* and *SNZ1,* were associated with very low levels of RNAP II, H3K4me3, H3K36me3, H3K79me3, and RNA in log cells, correlating with their lack of transcription in growing cells (Fig. 6b; Additional file 1: Figure S6B, S7D). Three days after culture inoculation (D3 Q), there was a dramatic and simultaneous rise in the levels of RNA, RNAP II, and the H3 PTMs associated with the two genes in the developing Q cells. RNAP II occupancy then decreased over time, while H3K4me3, H3K36me3 and H3K79me3 occupancies remained high. Interestingly, the levels of RNAP II CTD-Ser5P and CTD-Ser2-P associated with the two Q cell genes also peaked in day-3 and day-5 Q cells before dropping to lower levels in day-7 Q cells (Additional file 1: Figure S7A). This suggests that there was an extended period of time after diauxie in which these genes had high transcriptional activity, which was then followed by a gradual shut down of transcription. The data further support the view that the histone methylation marks were deposited on the Q genes during this period of active transcription and retained at high levels despite the decrease in transcription.

The common genes represented the largest subset of genes associated with transcripts in both log and Q cells (Fig. 4c, 3930 genes). A comparison of the enrichment of RNAP II and the H3 PTMs on this class of genes between log and day 7-Q cells revealed that the majority of these genes showed a decrease in RNAP II, H3K4me3, and H3K79me3 occupancy in Q cells, with a less dramatic difference in H3K36me3 occupancy occurring between the two cell types (Additional file 1: Figure S7C). Two common genes, *BAP2* and *OLE1*, were examined in more detail during Q cell development. Both genes were associated with approximately similar levels of transcripts in log and day-7 Q cells, but showed different patterns of RNAP II occupancy during Q cell development (Fig. 6c; Additional file 1: Figure S6C). At *BAP2*, RNAP II occupancy gradually decreased throughout the time course, similar to, but not as dramatic as the decrease in RNAP II occupancy at log-only transcribed genes (compare Fig. 6c and a; Rpb3-Myc; Additional file 1: Figure S7D). In contrast, the pattern of RNAP II occupancy at *OLE1* was more similar to the pattern seen at Q-only transcribed genes, with RNAP II occupancy rising over log levels in day-3 Q cells before falling off in day-5 and day-7 Q cells (Additional file 1: Figure S6C; 6b). The occupancy profiles of RNAP II CTD-Ser5P and CTD-Ser2-P on the two common genes were also somewhat heterogeneous. However, the general picture was that their levels increased at the two common genes in day-3 to day-5 Q cells before decreasing over the remainder of Q cell development (Additional file 1: Figure S7A).

The levels of H3K36me3 and H3K79me3 at both *BAP2* and *OLE1* during Q cell development remained similar to the levels seen in log cells, while the H3K4me3 occupancy profile at these genes was more heterogeneous (Additional file 1: Figure S6A, S6C compared to Fig. 6a and c). H3K4me3 occupancy at *BAP2* remained at log levels throughout the time-course, but H3K4me3 occupancy at *OLE1* dropped by ~2-fold between day-5 and day-7 Q cells, similar to the global decrease in the levels of this PTM observed at the majority of common genes (Additional file 1: Figure S6C, S7C, D). Together, the data suggest that the three H3 PTMs were deposited at the common genes during an earlier period of high transcriptional activity in both log and developing Q cells and then differentially retained in Q cells as transcription was gradually shut down.

### Development and maintenance of quiescence in mutants deficient for histone methylation

**Fig. 7:**
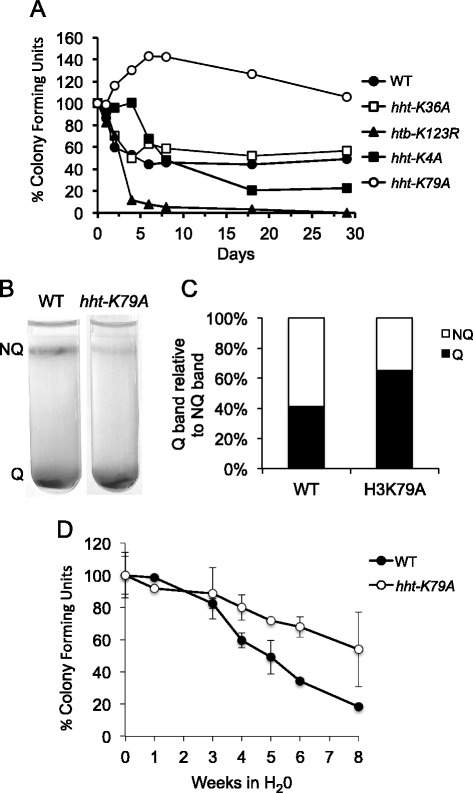
Histone modifications differentially affect the reproductive capacity of quiescent cells. **a**. Wild type cells (WT) and cells with mutations that abolish H2B ubiquitylation (*htb-K123R*), H3K4 methylation (*hht-K4A*), H3K36 methylation (*hht-K36A*), or H3K79 methylation (*hht-K79A*) were cultured in glucose-containing rich medium (YPD). Samples were removed from each culture at the diauxic shift (day 0) and at various times after glucose exhaustion for up to 30 days before spreading aliquots in triplicate onto YPD plates. The results are representative of a single experiment, and show the percentage of colony forming units relative to those at day 0, which was set as 100%. **b** Separation of wild type and *hht-K79A* cells on a Percoll gradient 7 days after inoculation into rich medium. **c** Relative proportions of quiescent and nonquiescent cells in 7-day cultures of wild type and *hht-K79A* strains. **d** Survival of wild type and *hht-K79A* quiescent cells incubated in water. The data represent the average with STD from two independent experiments. NQ, non-quiescent cell population; Q, quiescent cell population

To assess the biological significance of the histone methylation marks to cellular quiescence, we examined the reproductive capacity of yeast mutants deficient for H3K4, H3K36, or H3K79 methylation during growth into stationary phase (Fig. 7a). We also examined the reproductive capacity of a strain deficient for H2B ubiquitylation, as this modification regulates H3K4 and H3K79 methylation. The ability of stationary phase cells to re-enter the cell cycle after prolonged glucose deprivation is a measure of chronological aging - cells with a shortened chronological lifespan (CLS) have reduced reproductive capacity, while cells with an increased chronological lifespan have enhanced reproductive capacity. CLS is related to the proportion of Q cells in stationary phase cultures because NQ cells have a reduced ability to re-enter the cell cycle [14, 40]. A wild type strain, containing an approximately equal mix of both Q and NQ cells, showed an ~50% reduction in reproductive capacity 4 days after diauxie. An H2B ubiquitylation mutant showed a 90% decrease in reproductive capacity during the same period, correlating with a dramatic reduction in the number of quiescent cells formed in this strain [41]. An H3K4 methylation mutant also lost reproductive capacity [40], although the ability of this strain to form colonies decreased more slowly over time, so that by day-18 only 20% of the cells retained reproductive capacity. This decreased survival also correlated with an increased proportion of non-quiescent cells in the stationary phase culture (data not shown). In contrast, the absence of H3K36 methylation had no effect on the ability of stationary phase cells to re-enter the cell cycle. Surprisingly, an H3K79 methylation mutant displayed a 50% increase in colony formation after diauxie, which was then followed by a gradual drop in reproductive capacity over prolonged incubation. This striking phenotype prompted us to examine this mutant in more detail. In contrast to wild type cells, a higher proportion of quiescent cells were formed in the *hht-K79A* mutant (Fig. 7b, c). Moreover, when purified wild type and *hht-K79A* quiescent cells were assessed for their long-term ability to survive in water, the mutant Q cells showed an enhanced ability to re-enter the cell cycle (Fig. 7d). Together, the data suggest that different histone methylation marks have distinct roles in the development and maintenance of the quiescent cell population, with H3K4me and H3K79me having contrasting roles in the regulation of chronological lifespan.

## Discussion

Although quiescent cells are non-dividing, non-growing, and characterized by the global repression of transcription, these cells can rapidly respond to changes in their environment to resume growth and proliferation. In this study, we identified several features of yeast quiescent cells that could enable them to re-enter the cell cycle when conditions become favorable. First, quiescent cells contain a large cohort of RNAs that could become available for immediate translation. Second, these cells have a histone methylation landscape that is associated with transcriptionally active chromatin. Third, RNAP II remains associated with many genes in mature Q cells. We propose that the combined effect of these distinct features could enable quiescent cells to quickly turn-on the transcription and translation of growth promoting and proliferation genes.

### Transcription during the development of quiescence

The transcriptome analysis confirmed an earlier study that many gene-encoded RNAs are present in quiescent cells [17], although as previously reported, the total amount of RNA in these cells is lower than in growing cells [16]. The RNAs in quiescent cells include transcripts produced from Q-specific genes and genes common to both log and Q cells. Our analysis confirmed that there is a global shift in the distribution and levels of RNAP II on chromatin in mature Q cells compared to log cells [16], and further showed that Q cells have very low levels of both the initiating and elongating forms of the polymerase. However, we also found that after diauxie there appears to be a period of high transcriptional activity during the development of Q cells, with both the initiating and elongating forms of RNAP II retained in these cells for several days (Fig. 1e). An analysis of individual genes indicated that during this period, these active forms of RNAP II were associated with both Q-specific and common genes (Additional file 1: Figure S7A). This association corresponded to the rapid rise in RNA produced from Q-specific genes and could account for the continued transcription of the common genes as log cells transitioned from diauxie to quiescence. In contrast, active RNAP II was lost from log-only genes soon after glucose deprivation at diauxie, corresponding to their transcriptional repression. This suggests that as quiescent cells develop, there is a period in which RNAP II is simultaneously active at some genes (Q-specific and many common genes) and inactive at others (log-specific). Many of the transcripts produced during this period of activity then become stored or stabilized in mature quiescent cells and could promote their survival or re-entry into the cell cycle.

Although it was previously reported that in unseparated 7-day stationary phase cells RNAP II was poised at intergenic regions that are upstream of genes with roles in the rapid exit from quiescence [32], this bias was not observed in purified 7-day Q cells. Instead, in these mature Q cells, RNAP II was uniformly distributed across the coding region of both Q-specific and common genes and showed increased accumulation around the transcription end site (TES), which is the opposite profile to what was seen for RNAP II in actively growing cells (Fig. 3e). Moreover, this pattern was maintained in mature Q cells on genes that were only associated with RNAs in these cells (Additional file 1: Figure S3D). Thus, with the exception of genes expressed only in log cells, ongoing transcription may not be completely shut-off at the bulk of genes occupied by RNAP II in quiescent cells. Instead, the behavior of RNAP II may change during the development of quiescence, such that a lower frequency of transcription initiation, coupled with altered RNAP II processivity across the gene coding region, results in the peak of polymerase observed near the TES. Alternatively, the polymerase may not be actively engaged in transcription but retained on genes in Q cells as the consequence of aberrant polymerase release during transcription termination, which correlates with the presence of low levels of RNAP II Ser2-P CTD in the Q cell population. Altered termination could in turn inhibit transcription initiation, thereby forming a self-reinforcing feedback loop that ultimately down regulates transcription in Q cells.

### Chromatin in quiescent cells is associated with active histone methylation marks

It was previously reported that chromatin in Q cells is transcriptionally repressed as a consequence of the combined effects of increased nucleosome density and histone hypoacetylation at the TSS and coding region of many genes [16]. However, we found the histone modification landscape of quiescent cells is also notable for the retention of three histone methylation marks - H3K4me3, H3K36me3, and H3K79me3 - that are associated with transcriptionally active chromatin. Interestingly, these marks are also retained on the chromatin of unseparated stationary phase cells formed upon complete nutrient deprivation following transfer of cells into water [24], suggesting that they generally define the chromatin structure of non-growing cells. Unlike RNAP II, each of the H3 PTMs occupied similar locations on genes in growing and quiescent cells. H3K4me3 was present at the promoter and 5’ region of many genes in Q cells and could poise these genes for the initiation of transcription upon glucose restoration, similar to its postulated role at the promoters of genes in quiescent mammalian stem cells [2]. The coding regions of genes in quiescent cells also contained high levels of H3K36me3 and H3K79me3, marks associated with transcription elongation [42]. These modifications might also create a chromatin environment permissive for transcription under conditions that promote growth. Alternatively, rather than poising genes for activation, the marks might reinforce transcriptional repression in quiescent cells by recruiting HDACs or inhibiting histone exchange, two properties ascribed to the presence of H3K36me3 in gene coding regions in growing cells [43]. The modifications might play other roles as well, such as providing a chromatin template that is more permissive for DNA damage repair in quiescent cells, a feature associated with H3K79 methylation in growing cells [44, 45].

The three PTMs were associated with all three categories of genes in quiescent cells - Q-specific genes, genes common to log and Q cells, and log-specific genes. However, the mechanisms accounting for their presence on these genes might be different. It is likely that H3K4me3 was placed on Q-specific genes through its coupling to transcription during the post-diauxic period when RNAP II remains active. Because many common genes may continue to be transcribed during the same period, this could also account for the presence of H3K4me3 on this group of genes. Alternatively, the mark might be placed on both the common genes and log-specific genes during transcription in log cells, and then retained on these genes during the development of Q cells. The decreased correlation of H3K4me3 with RNAP II on genes in Q cells, along with the significant reduction in Set1 levels in post-diauxic cells, supports the latter scenario (Fig. 1c; Fig. 5a, b). It is unclear how H3K4me3 is retained on genes during the development of quiescence. In actively growing cells, it has been shown that H3K14 acetylation (H3K14ac) is required for the maintenance of global H3K4me3 levels via a mechanism whereby H3K14ac blocks the activity of the Jhd2 histone demethylase, which predominantly targets H3K4me3 for removal [46, 47]. However, in Q cells, H3K4me3 is retained on genes in the absence of H3K14ac (Fig. 1a; Additional file 1: Figure S1E). Although transcripts from genes encoding H3 demethylating enzymes are present in Q cells at levels equivalent to or even higher than those in log cells (Additional file 5: Table S5), it is not known if these enzymes are in fact present in Q cells. Alternatively, it is conceivable that the condensed chromatin structure of quiescent cells could inhibit their access to the modifications.

In contrast to H3K4me3, there was only a weak association between the presence of H3K36me3 and RNAP II in either log or Q cells (Fig. 5a). Instead, H3K36me3 correlated with H3, with a significantly stronger correlation seen in quiescent cells (Fig. 5a). Moreover, the levels of H3K36me3 on genes in both cell types were approximately the same (Fig. 2f). One interpretation of these results is that H3K36me3 marked nucleosomes, both those formed on Q-specific genes during the post-diauxic period and on the larger cohort of log-specific and common genes in log cells, were retained in developing Q cells as a consequence of decreased replication-dependent or –independent nucleosome turnover, coupled with the absence of demethylation. Interestingly, H3K4me3 levels on the large group of log-specific and common genes decreased approximately 2-fold in quiescent cells (Fig. 2f), which could be the consequence of dilution through replacement with unmodified H3 during the post-diauxic period. This suggests that H3K36me3 and H3K4me3 marked nucleosomes might be differentially turned over as quiescent cells develop. In support of this view, there was no correlation between the presence of H3K36me3 and H3K4me3 or between H3K4me3 and H3 across the genome in either log or Q cells (Fig. 5a).

Like H3K4me3 and H3K36me3, H3K79me3 is retained on the majority of genes in quiescent cells. However, unlike H3K4me2 and H3K36me2, H3K79me2 (and H3K79me1) is lost after diauxie (Fig. 1b). In contrast, all three H3K79 methyl species are present in nonquiescent cells. There is no known H3K79 demethylase that can counteract the Dot1 methyltransferase. However, it has been reported that H3K79me3 preferentially accumulates on “old” H3 proteins, which represent histones with a low-turnover rate [48]. The lengthy DNA replication cycle that occurs after diauxie suggests that the shift to H3K79me3 could be a consequence of the increased residence time of histones during this stage of Q cell development. An interesting corollary is that “old” H3-H4 tetramers might be preferentially segregated to Q cells that are formed during this period of DNA replication. Alternatively, a decreased rate of replication-independent histone turnover in these cells might also provide an increased pool of old H3 on which H3K79me3 is enriched. Together, the data suggest that the dynamics of histone turnover during the post-diauxic period might underlie the retention of all three H3 PTMs on genes in quiescent cells.

### Histone modifications differentially regulate cellular quiescence

The contrasting phenotypes exhibited by histone mutants deficient for the various PTMs when grown into stationary phase suggests that each histone modification has distinct roles in the establishment or maintenance of cellular quiescence. The decreased number of Q cells formed in mutants deficient for H2Bub1 suggests a role for this modification in promoting Q cell formation, potentially by preventing apoptosis from occurring in daughter cells [41]. Conversely, the increase in the proportion of Q cells formed in the absence of H3K79 methylation and the ability of the mutant quiescent cells to survive longer under conditions of extreme nutrient deprivation, suggest a role for this modification in promoting apoptosis in mother cells. In wild type cells, the natural loss of H2B ubiquitylation and the shift to H3K79me3 marked nucleosomes might together act as signals for the post-diauxic cell cycle arrest and entry into quiescence [36, 49].

In contrast to the loss of H2B ubiquitylation or H3K79 methylation, the loss of H3K36 methylation had no apparent effect on the formation or reproductive capacity of quiescent cells and thus does not influence chronological aging. Interestingly, mutations that eliminate H3K36 methylation result in a shorter replicative life span in yeast as the result of a decrease in transcriptional fidelity in aging cells [50]. Mutations that affect H3K79 methylation also decrease replicative life span [50], but have the opposite effect during chronological aging by promoting the formation of quiescent daughter cells. The contrasting effects of histone post-translational modifications on replicative and chronological life span suggest that different epigenetic mechanisms are used to control these two modes of cellular aging.

## Conclusions

In summary, quiescent yeast cells have molecular signatures that could ensure their long-term survival and ability to re-enter the cell cycle. These signatures include the presence of multiple transcripts encoding proteins important for growth and cell division, a histone modification landscape that favors new rounds of transcription, and a distinctive pattern of RNAP II occupancy across gene coding regions in quiescent cells. These signatures appear to be established both in growing cells and during the development of quiescent cells. As a consequence, similar to quiescent human stem cells, quiescent yeast cells are not inert, but appear highly poised to resume growth and proliferation when the environment becomes favorable.

## Methods

### Yeast strains, growth, and stationary phase cell fractionation

*S. cerevisiae* strains are listed in Additional file 1: Table S1. Cells were cultured at 30 °C in YPD + A medium (0.4 mg/mL adenine and 50 mg/mL ampicillin) and grown to an OD_600_ of 0.8 – 1.0 for log phase samples. Cells were grown through the diauxic shift into stationary phase and separated into quiescent (Q) and nonquiescent (NQ) populations at 3, 5, 7 and 14 days after inoculation following a published protocol [14]. Purified Q and NQ cells were washed in five volumes of Tris–HCl buffer, pelleted, and then treated with 20% TCA for Western blot analysis. Purified Q cells were fixed with 1% formaldehyde for chromatin immunoprecipitation assays. For measurement of the reproductive capacity of histone modification mutants grown into stationary phase, cells were grown in YPD + A medium at 30 °C. At intervals up to 30 days after culture inoculation, samples were removed and dilutions were spread in triplicate onto YPD plates. Colonies were counted 2–3 days after incubation of plates at 30 °C.

### Western blot analysis

TCA extracts were prepared from 10 OD_600_ units of log phase and separated quiescent and non-quiescent cells isolated from at least 2–3 independent experiments [51]. A sonication step of 4 × 10 s was added after glass-bead lysis and collection in 500 μL 5% TCA. 10 – 50 μg of protein were separated on 10 or 15% polyacrylamide gels, blotted to an Immobilon-P membrane, and incubated with the antibodies described in the following section.

### Antibodies

Western blots were incubated with the following antibodies: anti-FLAG (Sigma F3165: 1/15,000); anti-beta actin (Abcam ab8224: 1/3000–10,000); anti-Myc (Millipore 05–419, 9E10 monoclonal: 1/2000); anti-H2A (Active Motif 39235: 1/3000-10,000); anti-H2A.Z (Active Motif 39647: 1/1000-2000); anti-H2B (Active Motif 39237: 1/2500-10,000); anti-H3 (Active Motif 39163: 1/5000-7500; anti-H4 (Abcam ab7311: 1/1000); anti-H3K9/K14Ac (Millipore 06–599: 1/5000); anti-H3K9ac (Millipore 07–352: 1/5000); anti-H3K14ac (Millipore 07–353: 1/5000); anti-H4tetraAc (Millipore 06–886: 1/2500-6000); anti-H4K16ac (Active Motif 39929: 1/2000); anti-H3K56ac (Millipore 07-677-I: 1/1000-2000); anti-H3K4me1 (Millipore 07–436: 1/2000); anti-H3K4me2 (Active Motif 39913: 1/5000 and Abcam ab7766: 1/1000); anti-H3K4me3 (Active Motif 39159: 1/3000-5000); anti-H3K36me2 (Active Motif 39255: 1/5000); anti-H3K36me3 (Abcam ab9050: 1/1000-3000); anti-H3K79me1 (Abcam ab2886: 1/4000 and Active Motif 39145: 1/2000); anti-H3K79me2 (Millipore 04–835: 1/2000-3000); anti-H3K79me3 (Abcam ab2621: 1/7000-10,000); H14, anti-CTD-Ser5P (Covance MMS134R: 1/1000 and Active Motif 39750: 1/1000); H5, anti-CTD-Ser2P (Covance MMS-129R: 1/500-2000); anti-Gcn5 (Santa Cruz sc-9078: 1/500); anti-Rpd3 (Santa Cruz sc-6655: 1/500); anti-Hda1 (Santa Cruz sc-9077: 1/500); anti-Sir2 (Santa Cruz sc-6666: 1/500); anti-TBP (Abcam ab61411: 1/4000); and anti-Srb4 (Abcam ab63812: 1/5000).

### RNA quantitation

Measurement of transcript abundance was achieved by two-step RT-qPCR. Total RNA isolated by the hot phenol method [17] was converted to cDNA by reverse transcription. cDNA was then used as template for qPCR using gene-specific primer pairs. For analysis of mid-log phase transcript abundance samples were normalized to *ACT1.* For quantification of RNA abundance throughout stationary phase time course experiments, normalization to *ACT1* was not possible due to changes in *ACT1* transcription over the time course. Instead, two methods of normalization were carried out. One method used normalization of transcript abundance to total RNA levels, and the other used normalization to total RNA levels and normalization to an endogenous source (MS2) of RNA. While both methods produced similar gene-specific transcript profiles, the non-spiking method was employed due to ease of use and superior reproducibility.

### RNA-Seq analysis

Total RNA was isolated by the hot phenol method [17] from three biological replicates using 10 OD_600_ units of log phase cells and 7 day Q cells (RNA-seq), or from 1×10^7^ log phase cells or Q cells isolated 3, 5, and 7 days after culture inoculation. 5 μg of total RNA were treated with DNase (RQI DNase, Promega), and ERCC RNA Spike-In Control Mixes #1 and 2 were added before removal of the ribosomal RNA with the Ribo-ZeroTM Magnetic Gold Kit for yeast (Epicentre #MRZY1324). Libraries were prepared using the Ion Total RNA-Seq Kit v2 for Whole Transcriptome Libraries (Ion Torrent #4476290) using 250 ng of rRNA-depleted RNA. Sequencing was performed on the Ion Proton System in the University of New Mexico Comprehensive Cancer Center’s Analytical and Translational Genomics Shared Resource, and reads were mapped to the yeast genome.

### Chromatin immunoprecipitation

Thirty OD units of log cells and 40–50 OD units of purified 7-day Q cells were fixed in 1% formaldehyde for 20 min, quenched with 125 mM glycine for 5 min, washed in cold TBS, quick-frozen on dry ice and stored at −80 °C [52]. 1 mg of protein from whole cell lysates was added to FA lysis buffer to a total volume of 500 μl. The FA lysis buffer for anti-Myc ChIPs additionally contained 0.1% SDS. Lysates were incubated overnight at 4 °C with the following antibodies: 2.5 μl anti-H3 (Millipore 05–724); 2.5–4 μl anti-H3K4me3 (Active Motif 39159); 8 μl anti-H3K36me3 pre-bound to 60 μl Protein A beads (Abcam ab9050); 2 μl anti-H3K79me3 (Abcam ab2621); and 2.5 μl anti-Myc (Rpb3-Myc) (Millipore 05–724). After collection of immune complexes, the immunoprecipitates were sequentially digested with RNase A and Pronase, and the cross-links were reversed by incubation at 65 °C. DNA was extracted using a QiaQuick PCR Purification kit (Qiagen #28106). RNA Pol II CTD-Ser5P and CTD-Ser2P chromatin immunoprecipitations were performed as outlined in [53] and Stock *et al*. (Epigenesys.eu/en/protocols/chromatin-immunoprecipitation-chip/219-phospho-sensitive-chromatin-immunoprecipitation-of-rna-polymerase-II), with the following modifications. Twenty Microliter of Covance MMS-134R H14 or MMS-129R H5 antibodies were pre-bound to IgM agarose beads before overnight incubation with 700 μg of lysate. An additional high salt wash of 500 mM NaCL was included to elute the H14 immune complexes from IgM beads.

### ChIP-chip analysis

Two independent ChIP experiments were performed in log and 7-day Q cells using antibodies against Rpb3-Myc, H3K4me3, H3K36me3, and H3K79me3. The protocol used to amplify DNA was adapted from [54], as modified by the DiRisi and Rando labs. 7.5 μg of amplified ChIP and input DNAs from each experiment were hybridized at the same time to Affimetrix GeneChip^R^ *S. cerevisiae* 1.0R Tiling Arrays. The fragmentation, labeling, hybridization, and array processing were performed according to the Affymetrix Chromatin Immunoprecipitation Assay Protocol's User Guide in the Analytical and Translational Genomics Shared Resource at the University of New Mexico Comprehensive Cancer Center. Data analysis is described in Supplemental Methods.

## Additional files

Additional file 1: Figures S1-S7, Table S1, and Supplemental Methods. Supplemental figures S1-S7, supplemental table S1, supplemental methods, and figure legends for all supplemental figures and tables. (PDF 4.53 mb)
Additional file 2: Table S2. Lists of genes associated with H3K4me3, H3K36me3, H3K79me3, and RNAP II in growing and quiescent cells. (XLSX 32 kb)
Additional file 3: Table S3. Gene ontology categories for genes associated with RNAP II, H3K4me3, H3K36me3, and H3K79me3 in growing and quiescent cells. (XLSX 368 kb)
Additional file 4: Table S4. Association of Rpb3-Myc with intergenic regions in growing and quiescent cells. (XLSX 139 kb)
Additional file 5: Table S5. RNA-Seq data. (XLSX 492 kb)
Additional file 6: Table S6. Lists of genes associated with transcripts in growing and quiescent cells. (XLSX 72 kb)
Additional file 7: Table S7. Gene ontology categories for genes associated with transcripts in growing and quiescent cells. (XLSX 119 kb)

## References

[1] Valcourt JR, Lemons JM, Haley EM, Kojima M, Demuren OO, Coller HA (2012). Staying alive: metabolic adaptations to quiescence. Cell Cycle.

[2] Cheung TH, Rando TA (2013). Molecular regulation of stem cell quiescence. Nat Rev Mol Cell Biol.

[3] Joe AW, Yi L, Natarajan A, Le Grand F, So L, Wang J, Rudnicki MA, Rossi FM (2010). Muscle injury activates resident fibro/adipogenic progenitors that facilitate myogenesis. Nat Cell Biol.

[4] Uezumi A, Ikemoto-Uezumi M, Tsuchida K (2014). Roles of nonmyogenic mesenchymal progenitors in pathogenesis and regeneration of skeletal muscle. Front Physiol.

[5] Guenther MG, Levine SS, Boyer LA, Jaenisch R, Young RA (2007). A chromatin landmark and transcription initiation at most promoters in human cells. Cell.

[6] Freter R, Osawa M, Nishikawa S (2010). Adult stem cells exhibit global suppression of RNA polymerase II serine-2 phosphorylation. Stem Cells.

[7] Bernstein BE, Meissner A, Lander ES (2007). The mammalian epigenome. Cell.

[8] Bernstein BE, Mikkelsen TS, Xie X, Kamal M, Huebert DJ, Cuff J, Fry B, Meissner A, Wernig M, Plath K (2006). A bivalent chromatin structure marks key developmental genes in embryonic stem cells. Cell.

[9] Lien WH, Guo X, Polak L, Lawton LN, Young RA, Zheng D, Fuchs E (2011). Genome-wide maps of histone modifications unwind in vivo chromatin states of the hair follicle lineage. Cell Stem Cell.

[10] Woodhouse S, Pugazhendhi D, Brien P, Pell JM (2013). Ezh2 maintains a key phase of muscle satellite cell expansion but does not regulate terminal differentiation. J Cell Sci.

[11] Werner-Washburne M, Braun E, Johnston GC, Singer RA (1993). Stationary phase in the yeast Saccharomyces cerevisiae. Microbiol Rev.

[12] Gray JV, Petsko GA, Johnston GC, Ringe D, Singer RA, Werner-Washburne M (2004). “Sleeping beauty”: quiescence in Saccharomyces cerevisiae. Microbiol Mol Biol Rev.

[13] Herman PK (2002). Stationary phase in yeast. Curr Opin Microbiol.

[14] Allen C, Buttner S, Aragon AD, Thomas JA, Meirelles O, Jaetao JE, Benn D, Ruby SW, Veenhuis M, Madeo F, Werner-Washburne M (2006). Isolation of quiescent and nonquiescent cells from yeast stationary-phase cultures. J Cell Biol.

[15] Aragon AD, Rodriguez AL, Meirelles O, Roy S, Davidson GS, Tapia PH, Allen C, Joe R, Benn D, Werner-Washburne M (2008). Characterization of differentiated quiescent and nonquiescent cells in yeast stationary-phase cultures. Mol Biol Cell.

[16] McKnight JN, Boerma JW, Breeden LL, Tsukiyama T. Global Promoter Targeting of a Conserved Lysine Deacetylase for Transcriptional Shutoff during Quiescence Entry. Mol Cell. 2015;59:732–43. 10.1016/j.molcel.2015.07.014 PMC4560983 26300265

[17] Aragon AD, Quinones GA, Thomas EV, Roy S, Werner-Washburne M (2006). Release of extraction-resistant mRNA in stationary phase Saccharomyces cerevisiae produces a massive increase in transcript abundance in response to stress. Genome Biol.

[18] Galdieri L, Mehrotra S, Yu S, Vancura A (2010). Transcriptional regulation in yeast during diauxic shift and stationary phase. OMICS.

[19] Miles S, Li L, Davison J, Breeden LL (2013). Xbp1 directs global repression of budding yeast transcription during the transition to quiescence and is important for the longevity and reversibility of the quiescent state. PLoS Genet.

[20] Santos-Rosa H, Schneider R, Bannister AJ, Sherriff J, Bernstein BE, Emre NC, Schreiber SL, Mellor J, Kouzarides T (2002). Active genes are tri-methylated at K4 of histone H3. Nature.

[21] Krogan NJ, Kim M, Tong A, Golshani A, Cagney G, Canadien V, Richards DP, Beattie BK, Emili A, Boone C (2003). Methylation of histone H3 by Set2 in Saccharomyces cerevisiae is linked to transcriptional elongation by RNA polymerase II. Mol Cell Biol.

[22] Kouzarides T (2007). Chromatin modifications and their function. Cell.

[23] Li B, Carey M, Workman JL (2007). The role of chromatin during transcription. Cell.

[24] Mews P, Zee BM, Liu S, Donahue G, Garcia BA, Berger SL (2014). Histone methylation has dynamics distinct from those of histone acetylation in cell cycle reentry from quiescence. Mol Cell Biol.

[25] Li L, Miles S, Melville Z, Prasad A, Bradley G, Breeden LL (2013). Key events during the transition from rapid growth to quiescence in budding yeast require posttranscriptional regulators. Mol Biol Cell.

[26] Ng HH, Robert F, Young RA, Struhl K (2003). Targeted recruitment of Set1 histone methylase by elongating Pol II provides a localized mark and memory of recent transcriptional activity. Mol Cell.

[27] Krogan NJ, Dover J, Wood A, Schneider J, Heidt J, Boateng MA, Dean K, Ryan OW, Golshani A, Johnston M (2003). The Paf1 complex is required for histone H3 methylation by COMPASS and Dot1p: linking transcriptional elongation to histone methylation. Mol Cell.

[28] Laribee RN, Krogan NJ, Xiao T, Shibata Y, Hughes TR, Greenblatt JF, Strahl BD (2005). BUR kinase selectively regulates H3 K4 trimethylation and H2B ubiquitylation through recruitment of the PAF elongation complex. Curr Biol.

[29] Nakanishi S, Lee JS, Gardner KE, Gardner JM, Takahashi YH, Chandrasekharan MB, Sun ZW, Osley MA, Strahl BD, Jaspersen SL, Shilatifard A (2009). Histone H2BK123 monoubiquitination is the critical determinant for H3K4 and H3K79 trimethylation by COMPASS and Dot1. J Cell Biol.

[30] Heidemann M, Hintermair C, Voss K, Eick D (1829). Dynamic phosphorylation patterns of RNA polymerase II CTD during transcription. Biochim Biophys Acta.

[31] Dong L, Xu CW (2004). Carbohydrates induce mono-ubiquitination of H2B in yeast. J Biol Chem.

[32] Radonjic M, Andrau JC, Lijnzaad P, Kemmeren P, Kockelkorn TT, van Leenen D, van Berkum NL, Holstege FC (2005). Genome-wide analyses reveal RNA polymerase II located upstream of genes poised for rapid response upon S. cerevisiae stationary phase exit. Mol Cell.

[33] Hentrich T, Schulze JM, Emberly E, Kobor MS (2012). CHROMATRA: a Galaxy tool for visualizing genome-wide chromatin signatures. Bioinformatics.

[34] Schulze JM, Hentrich T, Nakanishi S, Gupta A, Emberly E, Shilatifard A, Kobor MS (2011). Splitting the task: Ubp8 and Ubp10 deubiquitinate different cellular pools of H2BK123. Genes Dev.

[35] Pokholok DK, Harbison CT, Levine S, Cole M, Hannett NM, Lee TI, Bell GW, Walker K, Rolfe PA, Herbolsheimer E (2005). Genome-wide map of nucleosome acetylation and methylation in yeast. Cell.

[36] Schulze JM, Jackson J, Nakanishi S, Gardner JM, Hentrich T, Haug J, Johnston M, Jaspersen SL, Kobor MS, Shilatifard A (2009). Linking cell cycle to histone modifications: SBF and H2B monoubiquitination machinery and cell-cycle regulation of H3K79 dimethylation. Mol Cell.

[37] Rutledge MT, Russo M, Belton JM, Dekker J, Broach JR (2015). The yeast genome undergoes significant topological reorganization in quiescence. Nucleic Acids Res.

[38] Schafer G, McEvoy CR, Patterton HG (2008). The Saccharomyces cerevisiae linker histone Hho1p is essential for chromatin compaction in stationary phase and is displaced by transcription. Proc Natl Acad Sci U S A.

[39] Xu Z, Wei W, Gagneur J, Perocchi F, Clauder-Munster S, Camblong J, Guffanti E, Stutz F, Huber W, Steinmetz LM (2009). Bidirectional promoters generate pervasive transcription in yeast. Nature.

[40] Walter D, Matter A, Fahrenkrog B (2014). Loss of histone H3 methylation at lysine 4 triggers apoptosis in Saccharomyces cerevisiae. PLoS Genet.

[41] Walter D, Matter A, Fahrenkrog B (2010). Bre1p-mediated histone H2B ubiquitylation regulates apoptosis in Saccharomyces cerevisiae. J Cell Sci.

[42] Wozniak GG, Strahl BD (1839). Hitting the ‘mark’: interpreting lysine methylation in the context of active transcription. Biochim Biophys Acta.

[43] Venkatesh S, Smolle M, Li H, Gogol MM, Saint M, Kumar S, Natarajan K, Workman JL (2012). Set2 methylation of histone H3 lysine 36 suppresses histone exchange on transcribed genes. Nature.

[44] Giannattasio M, Lazzaro F, Plevani P, Muzi-Falconi M (2005). The DNA damage checkpoint response requires histone H2B ubiquitination by Rad6-Bre1 and H3 methylation by Dot1. J Biol Chem.

[45] Tatum D, Li S (2011). Evidence that the histone methyltransferase Dot1 mediates global genomic repair by methylating histone H3 on lysine 79. J Biol Chem.

[46] Nakanishi S, Sanderson BW, Delventhal KM, Bradford WD, Staehling-Hampton K, Shilatifard A (2008). A comprehensive library of histone mutants identifies nucleosomal residues required for H3K4 methylation. Nat Struct Mol Biol.

[47] Maltby VE, Martin BJ, Brind’Amour J, Chruscicki AT, McBurney KL, Schulze JM, Johnson IJ, Hills M, Hentrich T, Kobor MS (2012). Histone H3K4 demethylation is negatively regulated by histone H3 acetylation in Saccharomyces cerevisiae. Proc Natl Acad Sci U S A.

[48] De Vos D, Frederiks F, Terweij M, van Welsem T, Verzijlbergen KF, Iachina E, de Graaf EL, Altelaar AF, Oudgenoeg G, Heck AJ (2011). Progressive methylation of ageing histones by Dot1 functions as a timer. EMBO Rep.

[49] Kim W, Choi M, Kim JE (2014). The histone methyltransferase Dot1/DOT1L as a critical regulator of the cell cycle. Cell Cycle.

[50] Sen P, Dang W, Donahue G, Dai J, Dorsey J, Cao X, Liu W, Cao K, Perry R, Lee JY (2015). H3K36 methylation promotes longevity by enhancing transcriptional fidelity. Genes Dev.

[51] Trujillo KM, Tyler RK, Ye C, Berger SL, Osley MA (2011). A genetic and molecular toolbox for analyzing histone ubiquitylation and sumoylation in yeast. Methods.

[52] Fleming AB, Kao CF, Hillyer C, Pikaart M, Osley MA (2008). H2B ubiquitylation plays a role in nucleosome dynamics during transcription elongation. Mol Cell.

[53] Komarnitsky P, Cho EJ, Buratowski S (2000). Different phosphorylated forms of RNA polymerase II and associated mRNA processing factors during transcription. Genes Dev.

[54] Bohlander SK, Espinosa R, Le Beau MM, Rowley JD, Diaz MO (1992). A method for the rapid sequence-independent amplification of microdissected chromosomal material. Genomics.

