# Supplementary material for: Distinct histone methylation and transcription profiles are established during the development of cellular quiescence in yeast
Source: BMC Genomics. 2017 Jan 26;18:107. doi: 10.1186/s12864-017-3509-9 (PMC5267397; doi:10.1186/s12864-017-3509-9)
Supplement: Additional file 1: Figures S1-S7, Table S1, and Supplemental Methods. — Supplemental figures S1-S7, supplemental table S1, supplemental methods, and figure legends for all supplemental figures and tables. (PDF 4.53 mb) [file 12864_2017_3509_MOESM1_ESM.pdf]

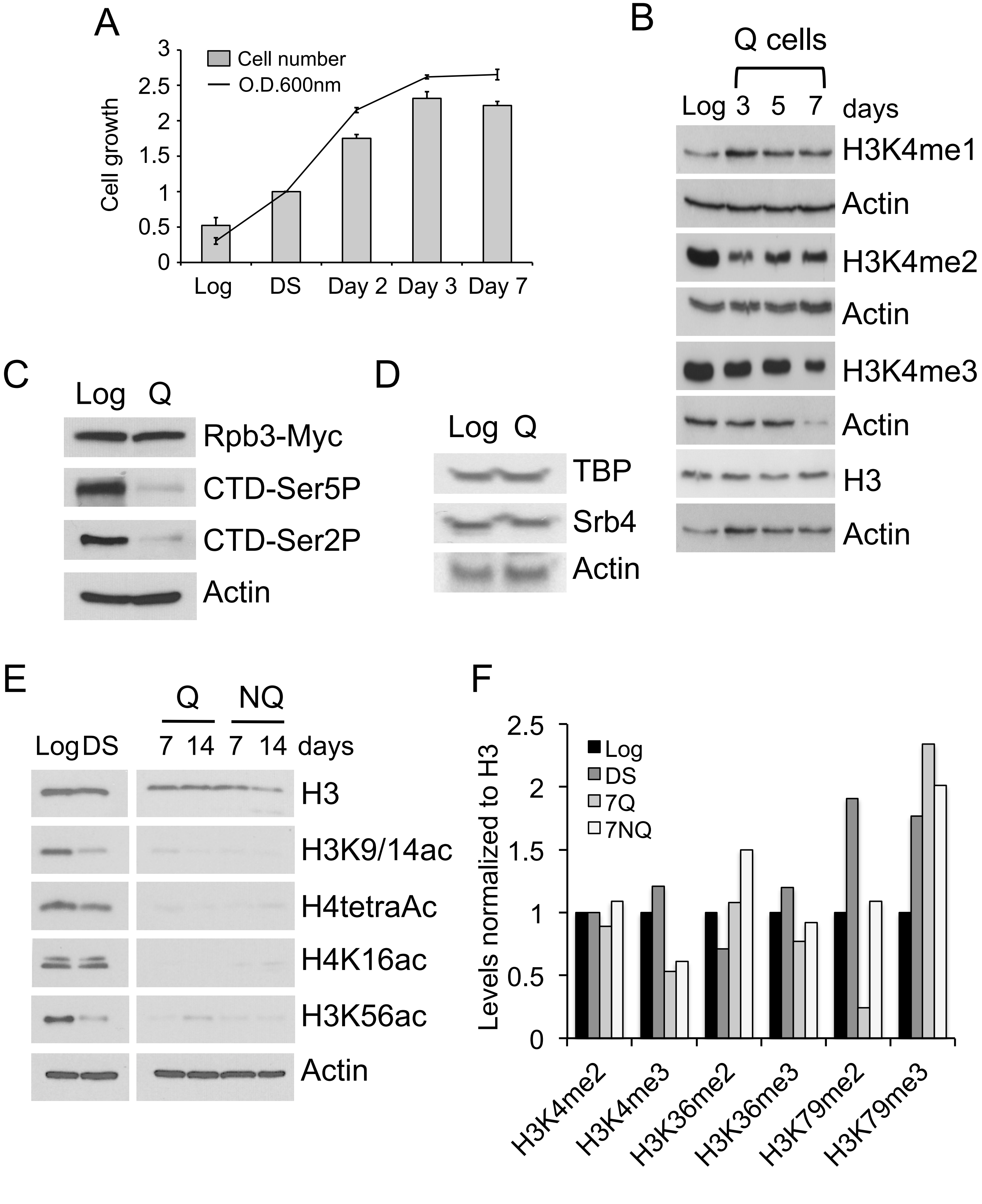
A
Cell number
O.D.600nm
Cell growth
3
2.5
2
1.5
1
0.5
0
Log
DS
Day 2
Day 3
Day 7
B
Q cells
Log 3 5 7 days
H3K4me1
Actin
H3K4me2
Actin
H3K4me3
Actin
H3
Actin
C
Log Q
Rpb3-Myc
CTD-Ser5P
CTD-Ser2P
Actin
D
Log Q
TBP
Srb4
Actin
E
Q NQ
Log DS 7 14 7 14 days
H3
H3K9/14ac
H4tetraAc
H4K16ac
H3K56ac
Actin
F
Levels normalized to H3
Log
DS
7Q
7NQ
2.5
2
1.5
1
0.5
0
H3K4me2
H3K4me3
H3K36me2
H3K36me3
H3K79me2
H3K79me3

Figure S1

A

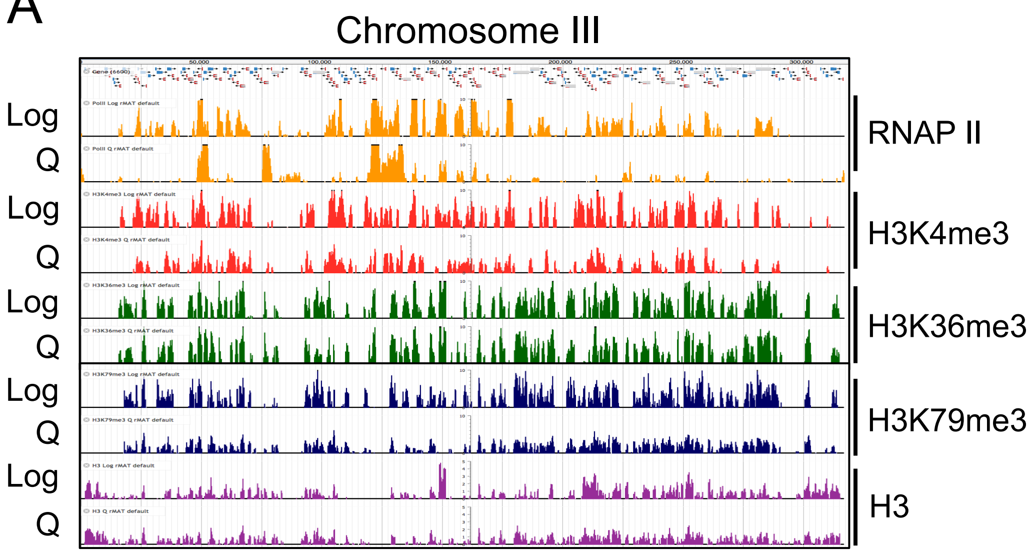

B

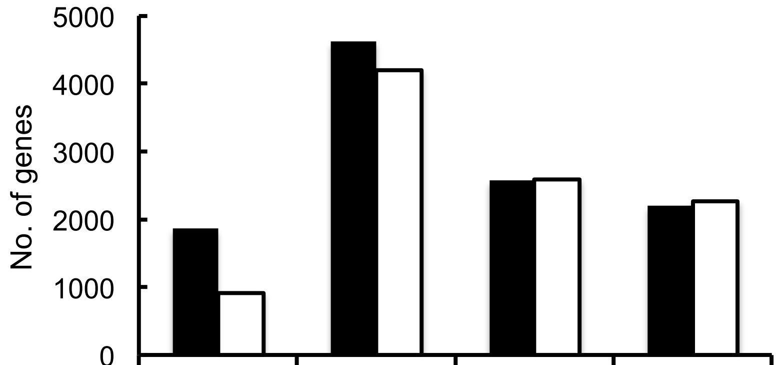

	RNAP II	H3K4me3	H3K36me3	H3K79me3
■ Log	1868	4623	2574	2203
□ Q	914	4199	2585	2266

C

RNAP II

GO terms: Log only
Ribosomes
Translation
RNA processing/maturation
Transcription
**GO terms: Q only**
Peroxisome
Fatty acid/lipid oxidation
Response to temperature
TCA cycle
**GO terms: Common**
Mitochondrion
Cell death
Response to temperature
Ion transport

H3K4me3

GO terms: Log only
Cell division
Cytoskeleton
Nucleolus
**GO terms: Q only**
Protein catabolism
Response to temperature/ stress
Glycogen metabolism
TCA cycle
**GO terms: Common**
Mitochondrion
Ribosome
Protein transport/localization
Protein catabolism
Transcription

H3K36me3

GO terms: Log only
Ribosome
Cytoskeleton
Cyclin-dependent kinase
**GO terms: Q only**
Response to temperature/ stress
TCA cycle
Peroxisome
Fatty acid oxidation
**GO terms: Common**
Nucleotide binding
Regulation of translation
Regulation of transcription
Budding
Protein transport/localization

H3K79me3

GO terms: Log only
Ribosome
Kinetochore
**GO terms: Q only**
Response to temperature/ stress
Fatty acid oxidation
Amino acid transport
**GO terms: Common**
Nucleotide/nucleoside binding
Golgi apparatus
Budding
Mitochondrion
Protein transport/localization

Figure S2

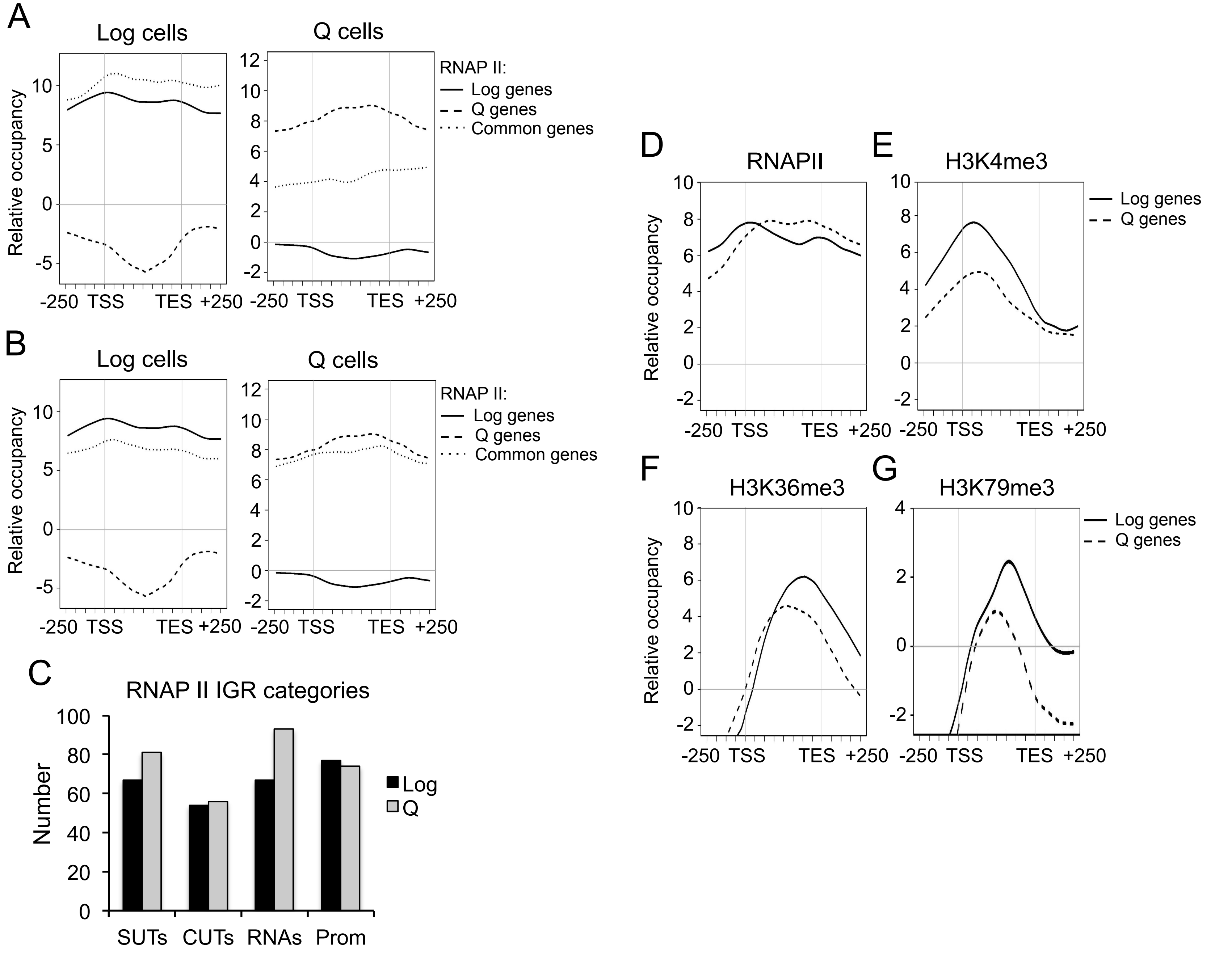
A
Log cells
Q cells
Relative occupancy
RNAP II:
Log genes
Q genes
Common genes
-250 TSS TES +250
B
Log cells
Q cells
C
RNAP II IGR categories
Number
Log
Q
SUTs CUTs RNAs Prom
D
RNAPII
E
H3K4me3
F
H3K36me3
G
H3K79me3

Figure S3

A

	Log	Q
All RNAs	6205	5105
ORFs	5402	4347
CUTs	189	120
SUTs	184	210
snRNAs	84	84

B

RNA

GO-Terms: Log cells only
RNA processing
ribosomes
regulation of translation
nuclear-cytoplasmic transport
mitosis
budding/cell polarity
spindle
DNA synthesis

GO-Terms: Q cells only
response to heat
peroxisome
protein catabolism
glucose/hexose metabolism
fatty acid oxidation
sporulation

GO-Terms: Common to Log & Q cells
transposition/DNA integration
protease activity/proteasome
ion transport
DNA recombination
DNA polymerase activity/general nucleases
ribosome/translation
transcription

Figure S4

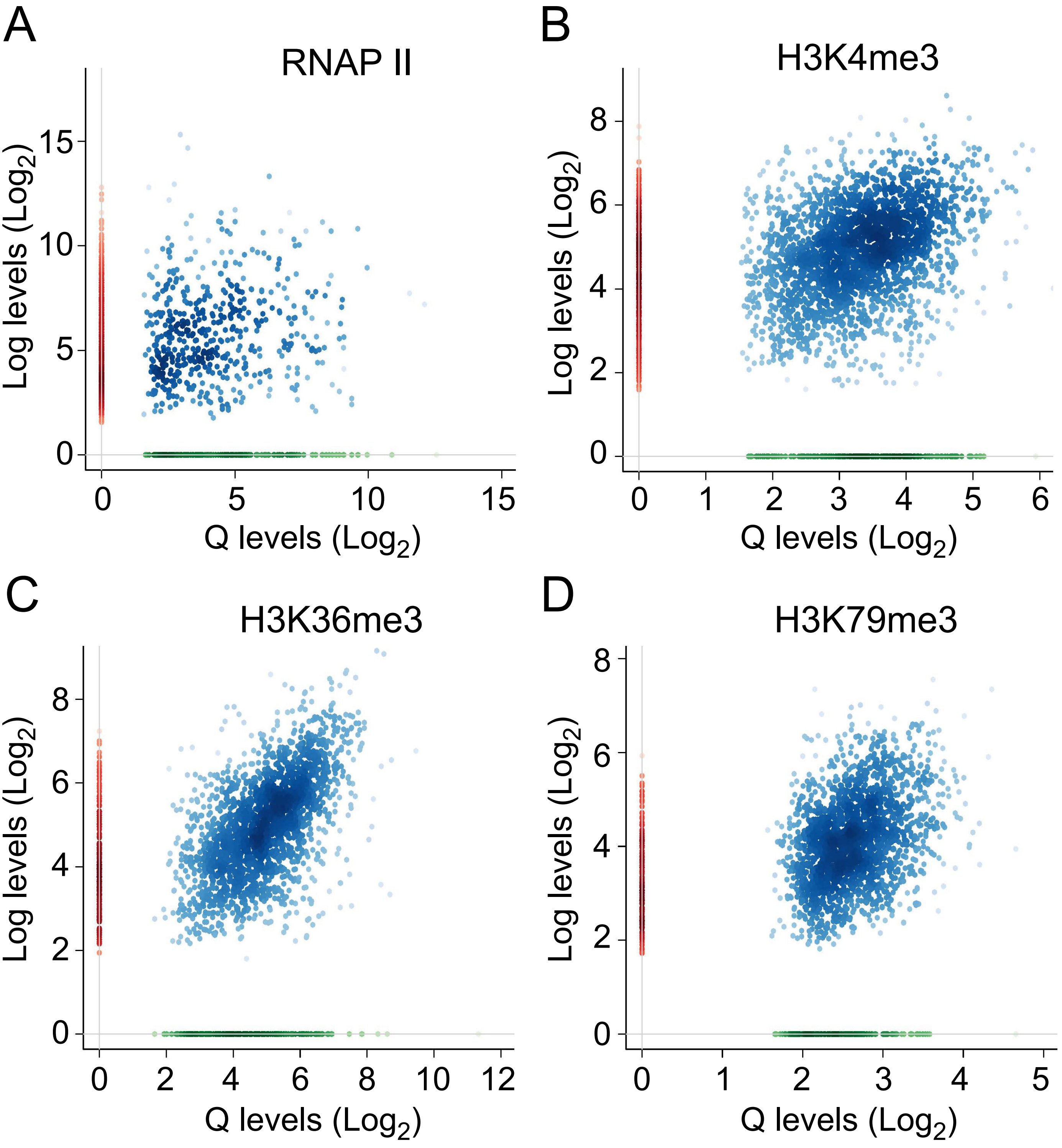

A
RNAP II
Log levels (Log_2)
Q levels (Log_2)
B
H3K4me3
Log levels (Log_2)
Q levels (Log_2)
C
H3K36me3
Log levels (Log_2)
Q levels (Log_2)
D
H3K79me3
Log levels (Log_2)
Q levels (Log_2)

Figure S5

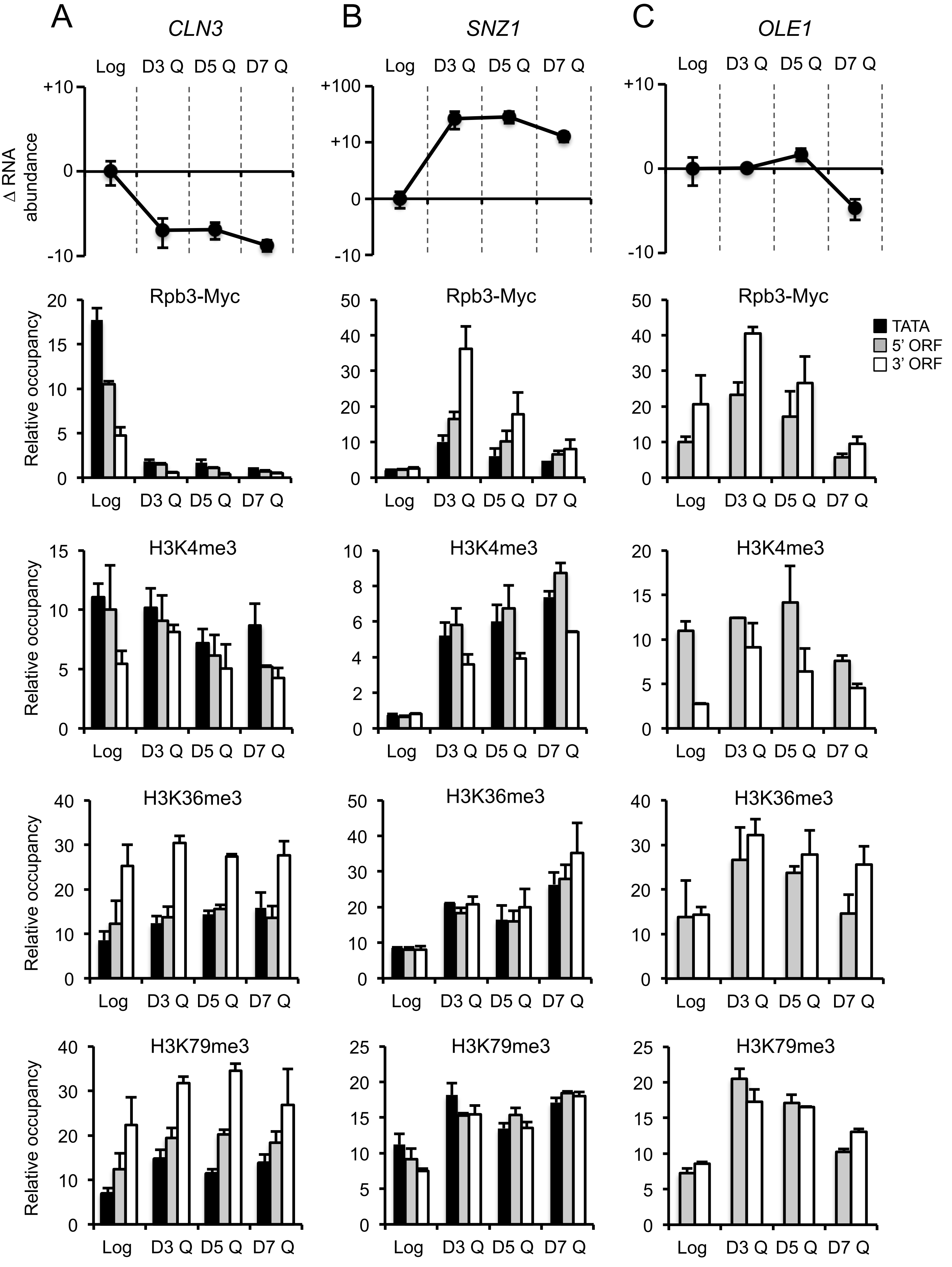

A
CLN3
B
SNZ1
C
OLE1
Log
D3 Q
D5 Q
D7 Q
Δ RNA abundance
Rpb3-Myc
H3K4me3
H3K36me3
H3K79me3
Relative occupancy
TATA
5' ORF
3' ORF

Figure S6

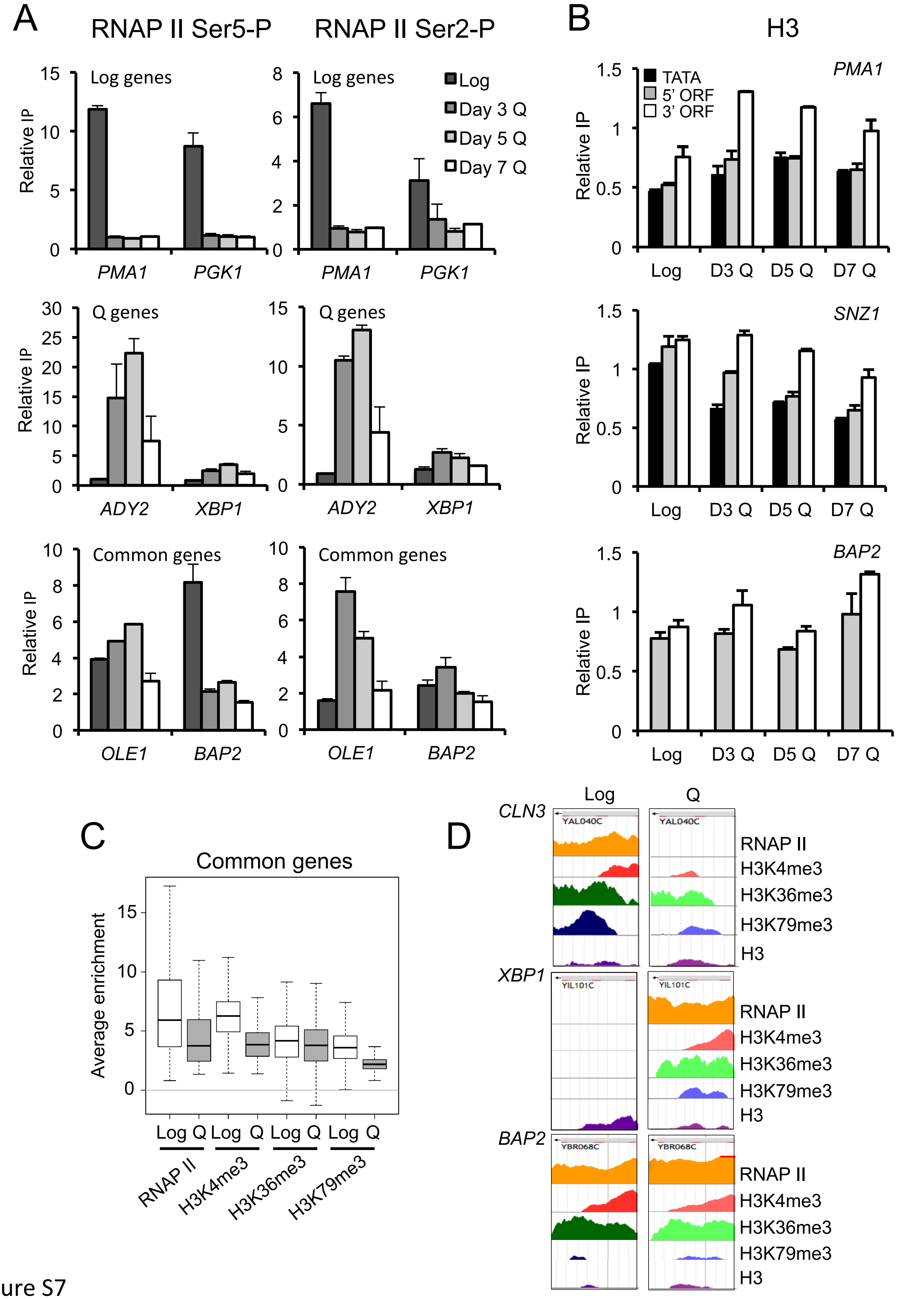

A
RNAP II Ser5-P
RNAP II Ser2-P
Log genes
Q genes
Common genes
Relative IP
Log
Day 3 Q
Day 5 Q
Day 7 Q
PMA1
PGK1
ADY2
XBP1
OLE1
BAP2
B
H3
TATA
5' ORF
3' ORF
PMA1
SNZ1
BAP2
Log
D3 Q
D5 Q
D7 Q
C
Common genes
Average enrichment
Log Q Log Q Log Q Log Q
RNAP II
H3K4me3
H3K36me3
H3K79me3
D
Log
Q
CLN3
XBP1
BAP2
YAL040C
YIL101C
YBR068C
RNAP II
H3K4me3
H3K36me3
H3K79me3
H3

Figure S7

Supplemental Figure Legends

Figure S1: Levels of histone methylation and acetylation, and preinitiation complex factors in quiescent cells.

A. Change in cell number and $O.D._{600}$ during growth from log phase (Log) into stationary phase relative to the diauxic shift (DS), which was set as 1. B. TCA lysates were prepared from log phase cells and purified Q cells isolated 3, 5, and 7 days after culture inoculation. Western blots were probed with antibodies against H3K4me1, me2, and me3. C-D. TCA lysates were prepared from log cells and 7-day Q cells and probed with antibodies against (C) Rpb3-Myc and RNAP II CTD phosphorylated on Serine 5 (CTD-Ser5P) and Serine 2 (CTD-Ser2P), and (D) TBP and Mediator subunit, Srb4. (E) TCA lysates were prepared from cells in log phase (Log), at the diauxic shift (DS), and in purified populations of quiescent (Q) and non-quiescent (NQ) cells isolated 7 and 14 days after culture inoculation. Western blots were probed with antibodies against acetylated histones and histone H3. The H3 and actin blots are those shown in Fig. 1A, as the same lysates were used for both analyses. Actin served as a loading control in all blots. F. Band intensities of H3 PTMs in the western blots shown in Fig. 1A were quantitated using the program, Image Studio. The band intensity for each PTM was normalized to the intensity of histone H3 in the same cell sample, and further normalized to the PTM/H3 ratio in log cells, which was set as 1.

Figure S2: Association of RNAP II, H3K4me3, H3K36me3, and H3K79me3 with genes in quiescent cells.

A. Genome browser images of RNAP II (Rpb3-Myc), H3K4me3, H3K36me3, H3K79me3, and H3 ChIP-chip data in log and 7-day Q cells. B. Number of genes marked with RNAP II and the three H3 methylations in log and 7-day Q cells. C. Gene ontology (GO) categories for unique and commonly marked genes in log and Q cells that are associated with RNAP II, H3K4me3, H3K36me3, and H3K79me3.

Figure S3: Gene association profiles of RNAP II and H3 PTMs in log and quiescent cells.

A, B. Average enrichment of RNAP II on common genes, log genes, and Q genes in log and Q cells. Profiles were defined using the top 25% RNAP II associated common genes in log cells (A). and in Q cells (B). The 250 bp regions upstream of the TSS (-250) and downstream of the TES (+250) are indicated. Solid line, log-specific RNAP II enrichment; dashed line, Q specific RNAP II

enrichment; dotted line, RNAP II enrichment on common genes. C. Divergent IGRs bound by RNAP II were characterized for the presence of SUTs, CUTs, and RNAs associated with yeast transposons (Table S4). Prom represents IGRs that do not contain these transcripts and defines RNAP II bound upstream of ORFs at presumptive gene promoters. D-G. Average enrichment of RNAP II (D), H3K4me3 (E), H3K36me3 (F), and H3K79me3 (G) on genes associated with RNA only in log cells (solid line) or only in Q cells (dashed line). The top 20% of genes in log or Q cells associated with RNA TPMs >10 were analyzed.

Figure S4: Categorization of ORF RNAs in quiescent cells.

A. The number of transcripts present in log and Q cells were divided into 4 categories: ORF (open reading frame); CUT (cryptic unannotated transcript); SUT (stable unannotated transcript); and snRNA (small nuclear RNA). B. Gene ontology (GO) categories for unique and commonly marked genes in log and Q cells that are associated with ORF RNAs.

Figure S5. Correlation between RNAP II and H3 methylations on genes in log and Q cells.

Scatterplots showing the correlation between RNAP II (A), H3K4me3 (B), H3K36me3 (C), and H3K79me3 (D) signals on genes in log and Q cells. Red = genes enriched only in log cells; green = genes enriched only in Q cells; blue = genes enriched in both log and Q cells.

Figure S6: Transcript, RNAP II, and H3 methylation profiles of individual genes during the development of quiescence.

Log cells and purified Q cells isolated 3, 5, and 7 days after culture inoculation were analyzed for transcript levels and Rpb3-Myc, H3K4me3, H3K36me3, and H3K79me3 occupancy at (A) a log-only expressed gene, *CLN3*; (B) a Q-only expressed gene, *SNZ1*; and(C) a gene expressed in both cell types, *OLE1*. Transcript levels represent the change in RNA abundance relative to levels in log cells. Rpb3-Myc and H3 PTM occupancies were determined by ChIP and qPCR at the TATA, 5' ORF or 3' ORF regions of each gene. Relative occupancy represents the IP/Input at each position relative to IP/Input at *TELV* (Rpb3-Myc) or to H3 IP/Input (H3K4me3, H3K36me3, H3K79me3). The data represent the average with STD from 2 independent experiments.

Figure S7: RNAP II CTD phosphorylation profiles of individual genes during the development of quiescence.

A. RNAP II CTD Serine 5 and Serine 2 phosphorylation occupancy at the 5' ORF (Ser5-P) or 3' ORF (Ser2-P) of genes associated with RNA only in log cells, only in Q cells, and in both cell types (common). B. H3 occupancy at genes associated with RNA only in log cells (*PMA1*), only in Q cells (*SNZ1*), or in both cell types (*BAP2*). Relative occupancy represents the IP/Input ratio at each gene relative to the IP/Input ratio at *TELV*. The data represent the average with STD from 2 independent experiments. C. Boxplots showing the average enrichment of RNAP II and the 3 H3 PTMs on genes associated with RNA in both log and 7-day Q cells (common genes). D. Genome browser view of RNAP II, H3K4me3, H3K36me3, H3K79me3, and H3 occupancy on genes associated with RNA only in log cells (*CLN3*), only in 7-day Q cells (*XBP1*), and in both log and 7-day Q cells (*BAP2*).

SUPPLEMENTAL TABLE LEGENDS

Table S1: List of *Saccharomyces cerevisiae* strains.

Table S2: Lists of genes marked with H3K4me3, H3K36me3, H3K79me3, and RNAP II in growing and quiescent cells. Genes marked only in growing cells (Log), only in quiescent cells (Q), or in both cell types (Common) were identified as described in **SUPPLEMENTAL METHODS**

Table S3: Gene ontology (GO) categories for genes associated with Rpb3-Myc (RNAP II), H3K4me3, H3K36me, and H3K79me3 in growing and quiescent cells. The DAVID functional annotation tool was used to identify categories of genes enriched only in log cells, only in 7-day Q cells, or in both cell types (Common), P value ≥ 10^{-4}.

Table S4: Association of Rpb3-Myc with intergenic regions in growing and quiescent cells. The association of Rpb3-Myc (RNAP II) with divergent intergenic regions (IGRs) in growing (log) and 7-day quiescent cells (Q) was determined as described in **SUPPLEMENTAL METHODS**. The table contains columns with information about ChIP enriched regions (CHERS) that includes chromosome, start and end position of the IGR, and score. Additional columns contain information on the percent coverage of the IGR, GCR (gene coding region), or "other" region with the RNAP II CHER. The feature with the highest percentage of coverage is listed in the

“dominant feature” column, followed by the % coverage of the IGR with CUTs, SUTs, and RNAs, and peak RNAP II enrichment in log and Q cells.

Table S5: RNA-Seq data. Data were collected from 3 independent RNA-seq experiments conducted on total RNA isolated from growing (log) and 7-day quiescent cells (Q). The table contains a column with gene information followed by columns with conversions to TPM numbers of the merged values for the 3 log and Q replicates. The last column contains the log2 ratio of the merged log TPM values divided by the merged Q TPM values.

Table S6: Lists of genes associated with transcripts in growing and quiescent cells. Genes associated with RNA only in growing cells (Log), only in 7-day quiescent cells (Q), or in both cell types (Common) were identified as described in **SUPPLEMENTAL METHODS**.

Table S7: Gene ontology (GO) categories for genes associated with transcripts in growing and quiescent cells. The DAVID functional annotation tool was used to identify categories of genes associated with transcripts only in growing cells (log), only in 7-day quiescent cells (Q), or in both cell types (Common), with P values $\geq 10^{-4}$.

SUPPLEMENTAL METHODS

CHIP-CHIP DATA PROCESSING

An adapted version of the Model-based Analysis of Tiling Arrays (MAT) algorithm for the R programming environment (rMAT) was used to normalize probe intensities (Droit et al., 2010). Each of the four ChIP DNAs (RNAP II, H3K36me3, H3K79me3 and H3K4me3) was normalized to input DNA, the resulting values were standardized, and biological replicates were averaged. Pearson correlation coefficients of the log2-transformed probe intensities were consistently above 0.9 between replicates. For each probe, the MAT scores were calculated as in Schulze *et al.* (2011) with a sliding window of 300 bp using a customized Perl script (available on GitHub, https://github.com/khokamp/Perl-scripts). The resulting scores were visualized across the genome using the JBrowse genome browser. ChIP enriched regions (CHERs) were computed using a customized Perl script (available on GitHub, https://github.com/khokamp/Perl-scripts). CHERs were computed using a 150 bp sliding window and were required to have a minimum average MAT score of 1.5.

Open reading frames (ORFs) were obtained from the *Saccharomyces* Genome Database (SGD) version R64.2.1. Only ORFs that were characterized as not 'Dubious' were used for the analysis. For RNAP II, H3K36me3 and H3K79me3, an ORF was defined as ChIP enriched when a CHER overlapped at least 50% of the ORF. For H3K4me3, the region around the transcription start site (TSS) (+200/-100 bp) was analyzed for ChIP enrichment. The overlap between CHERs and ORFs/TSS regions was computed using BEDTools (Quinlan and Hall, 2010). Using these criteria, ORFs that were ChIP enriched in either log phase or quiescent cells, but where ChIP binding was entirely absent in the other, were defined as log- or quiescent-specific, respectively. ORFs common to both log and quiescent cells showed ChIP enrichment in both cell populations (Table1).

ChIP condition	Number Log	% Log	Number Q	% Q	Number Common	% Common	Number Unenriched	% Unenriched
RNAP II	1219	21	265	4.5	649	11.2	3664	63.2
H3K4me3	664	11.4	240	4.1	3959	68.3	934	16.1
H3K36me3	216	3.7	227	3.9	2358	40.7	2996	51.7
H3K79me3	163	2.8	226	3.9	2040	35.2	3368	58.1

Table 1. Number of ORF regions with ChIP enrichment. An ORF was defined as ChIP-enriched if the ChIP enriched region (CHER) overlapping a given ORF had a minimum average MAT score of 1.5 and coverage of 50%. For H3K4me3, ChIP enrichment was measured in a region that encompassed -100 bp upstream and+200 bp downstream of the TSS, with 50% coverage.

Box plots, scatter plots and profile plots were all generated through the R statistical software (https://www.r-project.org/). For averaged gene profile plots the gene body and surrounding regions were organized into bins (40 for gene body, 20 each for upstream and downstream areas of 250 bp) to adjust for varying gene lengths. The Chromatra tool was utilized as described in Schulze *et al*. (2009).

DEFINITION OF IGRS

Intergenic regions (IGRs) that separate divergently transcribed ORFs were identified as described in Radonjic *et al*. (2005). Only ORFs with classification "verified" or "uncharacterized" were considered. GCRs represent ORFs. Any other regions in the genome not covered by IGRs or GCRS were classified as "other". The percentage coverage of Rpb3-Myc in IGRs, GCRs, and "other" regions was calculated from the Rpb3-Myc CHERs. IGR regions bound by Rpb3-Myc were based on 50% coverage of IGR and 50% coverage of adjacent GCR.

RNA-SEQ DATA PROCESSING

Reads were aligned and then assigned to genes in the yeast genome using a Perl script (available on GitHub, https://github.com/khokamp/Perl-scripts). The Pearson correlation coefficients of the log2-transformed read counts for the three log or Q cell replicates were consistently above 0.96. Read counts were transformed into TPMs (Transcript Per Million) following the algorithm described in Li *et al*. (2010). TPM values from replicates were merged using the mean, and the ratios between log and Q cells were calculated as the difference on the log2 scale of the merged values. Density plots of the log2-transformed TPM values showed a bimodal distribution, and the minimum between the two peaks in log cells was used to determine a threshold separating expressed genes from background noise (Fig. 1). Transcripts with TPM ≥2 were used for analysis.

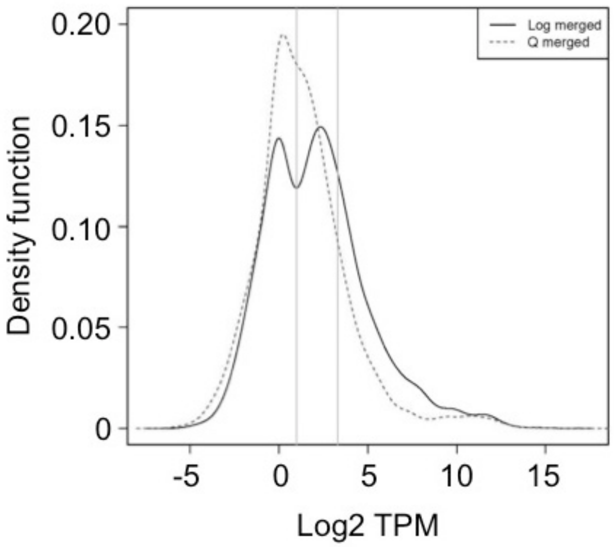

Figure 1: Density plots of log2-transformed TPM values.

SUPPLEMENTAL REFERENCES

Droit, A, Cheung, C, and Gottardo, R. 2010. rMAT – an R/Bioconductor package for analyzing ChIP-chip experiments. *Bioinformatics* 1; **26**:678-9.

Li B, Ruotti V, Stewart RM, Thomson JA, Dewey CN. 2010. RNA-Seq gene expression estimation with read mapping uncertainty. *Bioinformatics* **26**: 493-500.

Schulze JM, Jackson J, Nakanishi S, Gardner JM, Hentrich T, Haug J, Johnston M, Jaspersen SL, Kobor MS, Shilatifard A. 2009. Linking cell cycle to histone modifications: SBF and H2B monoubiquitination machinery and cell-cycle regulation of H3K79 dimethylation. *Mol Cell* 35: 626-641.

Quinlan, AR, Hall, IM. 2010. BEDTools: a flexible suite of utilities for comparing genomic features. *Bioinformatics* **26**:841-842.

Table S1: *Saccharomyces cerevisiae* strains

Strain	Genotype
**FY406**	MATa, (hta1-htb1)Δ::LEU2, (hta2-htb2)Δ::TRP1, his3Δ200, leu2Δ1, trp1Δ, ura3-52, <pSAB6 (HTA1-HTB1::URA3)> <pZS145 (HTA1-Flag-HTB1::HIS3)>
**YCH406**	MATa, (hta1-htb1)Δ::LEU2, (hta2-htb2)Δ::TRP1, his3Δ200, leu2Δ1, trp1Δ, ura3-52, RPB3-9Myc::hphNT1, <pSAB6 (HTA1-HTB1::URA3)> <pZS145 (HTA1-Flag-HTB1::HIS3)>
**YCY1**	MATa, (hta1-htb1)Δ::LEU2, (hta2-htb2)Δ::TRP1, his3Δ200, leu2Δ1, trp1Δ, ura3-52, DOT1-9myc::KANMX, <pSAB6 (HTA1-HTB1::URA3)> <pZS145 (HTA1-Flag-HTB1::HIS3)>
**YCY5**	MATa, (hta1-htb1)Δ::LEU2, (hta2-htb2)Δ::TRP1, his3Δ200, leu2Δ1, trp1Δ, ura3-52, SET1-9myc::KANMX, <pSAB6 (HTA1-HTB1::URA3)> <pZS145 (HTA1-Flag-HTB1::HIS3)>
**YCY6**	MATa, (hta1-htb1)Δ::LEU2, (hta2-htb2)Δ::TRP1, his3Δ200, leu2Δ1, trp1Δ, ura3-52, SET2-9myc::KANMX, <pSAB6 (HTA1-HTB1::URA3)> <pZS145 (HTA1-Flag-HTB1::HIS3)>
**YAF1**	MATa, hhf2-hht2::NAT, hta1-htb1::HPH, hht1-hhf1::KAN, hta2- htb2::NAT, ura3-52, trp1Δ2, leu2-3,-112, his3-11, ade2-1, can1-100; <pRS315 (HTA1-HTB1, HHT1-HHF1::LEU2)>
**YAF3**	MATa, hhf2-hht2::NAT, hta1-htb1::HPH, hht1-hhf1::KAN, hta2- htb2::NAT, ura3-52, trp1Δ2, leu2-3,-112, his3-11, ade2-1, can1-100; <pRS315 (HTA1-HTB1, hht1-K4A-HHF1::LEU2)>
**YAF9**	MATa, hhf2-hht2::NAT, hta1-htb1::HPH, hht1-hhf1::KAN, hta2- htb2::NAT, ura3-52, trp1Δ2, leu2-3,-112, his3-11, ade2-1, can1-100; <pRS315 (HTA1-HTB1, hht1-K36A-HHF1::LEU2)>
**YAF11**	MATa, hhf2-hht2::NAT, hta1-htb1::HPH, hht1-hhf1::KAN, hta2- htb2::NAT, ura3-52, trp1Δ2, leu2-3,-112, his3-11, ade2-1, can1-100; <pRS315 (HTA1-HTB1, hht1-K79A-HHF1::LEU2)>
**YAF97**	MATa, hhf2-hht2::NAT, hta1-htb1::HPH, hht1-hhf1::KAN, hta2- htb2::NAT, ura3-52, trp1Δ2, leu2-3,-112, his3-11, ade2-1, can1-100; <pRS315 (HTA1-htb1-K123R, HHT1-HHF1::LEU2)>
**YAF105**	MATa, hhf2-hht2::NAT, hta1-htb1::HPH, hht1-hhf1::KAN, hta2- htb2::NAT, ura3-52, trp1Δ2, leu2-3,-112, his3-11, ade2-1, can1-100; <pRS315 (HTA1-HTB1, hht1-K4A/K79A-HHF1::LEU2)>
